# Model Protective Films on Cu-Zn Alloys Simulating the Inner Surfaces of Historical Brass Wind Instruments by EIS and XPS

**DOI:** 10.3389/fchem.2020.00272

**Published:** 2020-04-15

**Authors:** Marzia Fantauzzi, Bernhard Elsener, Federica Cocco, Cristiana Passiu, Antonella Rossi

**Affiliations:** ^1^Dipartimento di Scienze Chimiche e Geologiche, Università degli Studi di Cagliari, Cagliari, Italy; ^2^INSTM, University of Cagliari Research Unit (UdR), Cagliari, Italy; ^3^Institute for Building Materials, ETH Zurich, ETH Hönggerberg, Zurich, Switzerland; ^4^Laboratory for Surface Science and Technology, Department of Materials, ETH Zurich, Zurich, Switzerland

**Keywords:** brass alloys, nanostructured surface film, neutral solutions, electrochemical impedance spectroscopy (EIS), X-ray photoelectron spectroscopy (XPS) and scanning electron microscopy (SEM)

## Abstract

The present work focuses on the characterization of brass surfaces after contact with artificial saliva solution at pH 7.4 and phosphate buffer solution at pH 7 simulating two extreme conditions that might occur when playing ancient brass wind instruments in the context of historically informed performance practice. The composition and the morphology of the film formed following the contact with the solutions for 1, 3, and 16 h were investigated by *ex situ* X-ray photoelectron spectroscopy (XPS) and scanning electron microscopy (SEM) to shed a light on the surface changes upon time. *In situ* electrochemical impedance spectroscopy (EIS) was used to study the mechanism of corrosion and protection of the alloys. The results could be interpreted using a reliable equivalent electrical circuit; they provided evidence that the alloys behave differently when in contact to the various solutions. In saliva solution the formation on the brass surface of a thick surface film was observed, composed of crystallites of about 200 nm size mainly composed of CuSCN and Zn_3_(PO_4_)_2_. This layer hinders the alloy dissolution. The contact of the alloys with the buffer solution originated a much thinner layer composed of Cu_2_O, ZnO, and a small amount of Zn_3_(PO_4_)_2_. This film is rapidly formed and does not evolve upon time in a protective film.

## Introduction

This work is part of a larger project whose overall goal was the development of an electrochemical sensor to understand the mechanism of corrosion and to monitor its evolution over time on brass musical instruments of the nineteenth and twentieth century^1^. The brass wind instruments collection under investigation is well-conserved in the Burri Museum in Bern (Switzerland) and it contains more than 1,200 artifacts. Some of them are going to be used in concerts in the context of the “historically informed performance practice” (HIP) (Butt, 2002), which is a dominant trend in contemporary musical practice that intends to play restored original period instruments in concerts. To verify the efficiency of preventive conservation measures and to obtain information on the corrosion state and rate inside the instruments, non-destructive electrochemical techniques were chosen. The goal was achieved using a miniaturized electrochemical cell that could be used outside but especially inside the tuning slides of the artifacts (Elsener et al., 2016b). Firstly, the work (Elsener et al., 2016b) has involved the development of the electrochemical sensor and subsequently its calibration; afterward the sensor has been applied on reference materials and then used on the historical brass musical instruments (Elsener et al., 2016a). Unfortunately, the surface state inside the tuning slides is unknown and it is difficult to find a technique able to characterize for instance the inside surface of the trumpets.

In our previous work (Cocco et al., 2016a) the electrochemical behavior of a series of brasses (Zn content from 18 to 38 wt. %) was investigated after contact with two neutral solutions: artificial saliva solution and diluted pH = 7 phosphate buffer solution at the open circuit potential (OCP). The first one was chosen as an aggressive medium. The composition of natural saliva may differ considerably and can be affected by the type and intensity of stimulation, diet, age, time of day, diseases, and pharmacological agents. Hence, an exact replica of human saliva is hardly possible. Different formulations have been developed for different purposes. Here the Tani-Zucchi artificial saliva has been chosen because its composition is very close to the actual conditions existent in a cavity media when considering the inorganic components (Tani and Zucchi, 1967). The pH 7-phosphate solution was chosen as a mild exposure condition. The results showed that the corrosion resistance increases with increasing exposure time. Moreover, brasses with high zinc content showed higher dissolution rates for both solutions. As far as the samples in contact with saliva solution the corrosion rate decreased from 60 to 0.5 μm/year after 16 h of exposure, while in the less aggressive phosphate buffer solution it decreased only by a factor of two, from 3 to 5 μm/year (1 h) to 1.5–2.5 μm/year (16 h). This difference could be ascribed to the different surface films: a thicker and more protective film composed of CuSCN and zinc phosphate was formed upon contact with the artificial saliva solution whereas a nanometer thick film was formed in the pH 7-phosphate solution made mainly of copper and zinc oxides.

This work is focused on obtaining a deeper insight into the corrosion and protection mechanism of brass alloys (Zn content 18–37%) when in contact to neutral solutions. It exploits the electrochemical impedance spectroscopy (EIS), a powerful tool in corrosion science for following the electrochemical behavior of metals exposed to different electrolytes. Moreover, the EIS is a non-destructive technique since the application of a small AC signal during the measurement does not alter the electrochemical properties (Barsoukov and Macdonald, 2018). An insight on the composition of surface films was gained by XPS/XAES surface analysis to contribute to explain their stability, the dissolution mechanism and the corrosion behavior. The occurrence of corrosion phenomena due to the exposure to the different electrolytes and the morphology of the different oxide films were also examined by optical- and scanning electron microcopies (SEM).

## Materials and Methods

### Materials and Surface Preparation

Experiments were carried out on five different brass alloys with a zinc content ranging from 18 to 38 wt. % Zn. The composition of the alloys, determined by XRF, is reported in Cocco et al. (2016a). The alloys CuZn18, CuZn28, CuZn35Pb1, and CuZn38Pb2 were produced according a procedure that allows obtaining compositions and metallurgical structures as close as possible to alloys used in the nineteenth century (Von Steiger et al., 2013). CuZn37 was a standard alloy purchased from Goodfellow Cambridge Ltd., UK. Thin sheets, about 0.5 mm thick, of the brass alloys were mechanically polished using a sequence of abrasive SiC papers down to 4,000 mesh (Struers, Ballerup—DK) using ethanol as cooling lubricant during grinding. The samples were mirror-like polished with a DP Dur cloth (Struers) using diamond pastes up to 1 μm, rinsed with ethanol and dried using an argon stream.

### Electrochemical Impedance Spectroscopy

The EIS measurements were performed on the mechanically polished samples immersed in artificial saliva (Tani and Zucchi, 1967) and in phosphate buffer solutions (1:10) at pH 7.4. The EIS analyses were conducted using a potentiostat/galvanostat VersaSTAT3 (Ametek, Inc., Princeton Applied Research, USA) connected to a three-electrode electrochemical cell. A saturated calomel electrode (0.241 V vs. NHE at 25°C, SCE) was chosen as reference electrode and all the measured potentials are referred to its value. A platinum mesh was used as counter electrode. The EIS measurements were carried out over a frequency range from 10 kHz to 0.01 Hz with 7 points per decade using AC amplitude of 5 mV. The EIS spectra were acquired after 1, 3, and 16 h exposure to both solutions. All measurements were carried out at ambient temperature (25 ± 2°C) with the solutions open to air.

### X-Ray Photoelectron Spectroscopy (XPS)

Surface analyses were performed using a Theta Probe spectrometer (Thermo Fisher Scientific, East Grinstead, UK). The residual pressure in the main chamber during the acquisition was lower than 10^−7^ Pa. The XPS spectra were collected using a 400 μm monochromatic beam (AlKα source 1486.6 eV) operated at 4.7 mA and 15 kV (70 W). The analyzed area is estimated to be 0.5 mm^2^ (Passiu et al., 2017). The average emission angle is 53° while the angle between the source and the analyser axis is 67.38°. Survey and high-resolution (HR) spectra were acquired in fixed analyzer transmission mode (FAT) setting the pass energy equal to 200 and to 100 eV, respectively, selecting the standard lens mode. The full-width at half-maximum (FWHM) of the Ag3d_5/2_peak acquired in the same conditions used for the HR spectra was 0.84 eV. The linearity of the binding energy (BE) scale was performed according to ISO 15472:2010 with an accuracy of ±0.1 eV. The BE values were referenced to the aliphatic carbon at 285.0 eV. Data were acquired under computer control (Avantage v. 3.45). Three different areas were analyzed on each sample. BE values and atomic percentages are reported in this work as mean values on three points with their corresponding standard deviations. The curve fitting procedure was performed using CasaXPS software (v2.3.16, Casa Software Ltd., Wilmslow, Cheshire, UK); the background was subtracted according to the Shirley-Sherwood background subtraction routine (Shirley, 1972) and then Gaussian and Lorentzian product functions were used for curve fitting. The quantitative analysis of brasses was performed on the basis of the integrated intensity using the first-principle approach (Briggs and Grant, 2003) under the assumption that the sample was homogeneous. The peak areas of each element were corrected for the respective sensitivity factor calculated taking into account Scofield's photoionization cross-sections (Scofield, 1976), the asymmetry factors (Reilman et al., 1976), the analyser transmission function T(E_i_) of the instrument (Fantauzzi et al., 2012), and the inelastic mean free paths (IMFP). The IMFP was calculated using the equation proposed by Tanuma et al. (2003). The accuracy of the calculated atomic concentrations is estimated to be ±10%. The sampling depth is estimated to be about 4 nm. In the following the authors will refer to thick film and thin films meaning films that are thicker and thinner than the sampling depth, respectively.

Quantitative analysis of the brass surfaces exposed to the phosphate buffer solution was also performed according to the three-layer model (Rossi and Elsener, 1992; Cocco et al., 2016b) and the thickness and composition of the surface layer together with the composition of the metal phase beneath the film could be calculated from a single XPS/XAES measurement. Since in the samples exposed to saliva solution the signals due to metallic copper and zinc were not detectable, the three-layer model was not appropriate and was not applied.

### Scanning Electron Microscopy (SEM)

Scanning Electron Microscopy (SEM) was used to observe the surface of the brass alloys before and after the EIS measurements. The instrument used in this work was a Zeiss Ultra-55 (Carl Zeiss, Feldbach, Switzerland) equipped with InLens, SE2 (Everhart-Thornley Secondary Electron) e EsB (energy selective backscattered) detectors but only the InLens and SE2 were used in this work. The applied beam potential was 5 kV.

## Results

### SEM Investigations

**Figure 1:**
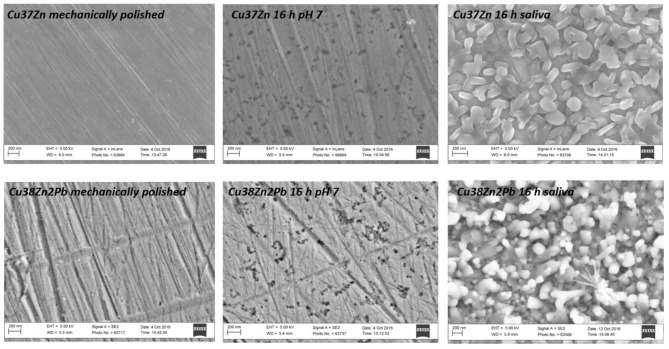
SEM images acquired on the Cu37Zn and Cu38Zn2Pb alloys following the mechanical polishing procedure, after 16 h of exposure to the phosphate buffer and to the artificial saliva solutions.

The surface morphology of brass samples was investigated by SEM analysis before and after the contact with the test solutions to monitor the surface modifications over the exposure time. Figure 1 shows the SEM images acquired on two different brasses: Cu37Zn and Cu38Zn2Pb before and after 16 h of exposure to the buffer and the artificial saliva solutions. The surface prior to contact showed thin scratches due to the mechanical polishing and no particles were found on it. Some white spots were observed, probably due to a residue of the diamond paste (diamond particles with an average size of 0.25 μm) used in the last step of the polishing procedure. After the contact with the buffer solution several black spots were visible at the surface of the alloy and they might be due to localized corrosion or dezincification areas. The surface of the alloy after the exposure to the saliva solution was homogeneously covered by particles of about 0.2 μm in size; in contrast to the buffer solution no scratches were detectable on the surface of the metal.

### Electrochemical Impedance Spectroscopy (EIS)

**Figure 2:**
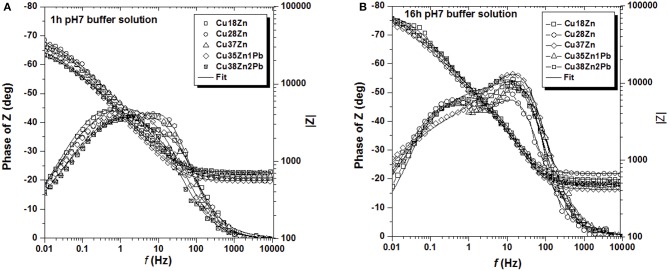
Bode plots of all the brass alloys in contact with the phosphate buffer pH 7 for 1 h **(A)** and for 16 h **(B)**.

The electrochemical behavior of brass in contact with the test solutions was investigated by EIS at the open circuit potential (OCP), the values can be seen in Tables 1, 2. The change of the OCP with time was in agreement with a previous paper (Cocco et al., 2016a). The impedance spectra of all brass alloys after 1 and 16 h immersion in the phosphate buffer solution are shown in Figure 2. Whereas, there is no significant difference among the brass alloys, a clear evolution of the spectra with time can be seen (Figure 2), indicating an increase of the impedance at low frequencies and the more pronounced appearance of two maxima in the phase angle after 16 h (Figure 2B). The Nyquist plots (Figures S.3, S.4) showed only one depressed semicircle. The extrapolation to the x-axis at the low frequency end of the EIS data indicates the polarization resistance Rp.

**Table 1:** Calculated fitting parameters for brass alloys after 1, 3, and 16 h of immersion in phosphate buffer solution; the standard deviation in percentage of the value is obtained from three replica measurements and is reported in parentheses.

	**Time (h)**	**OCP (mV)**	**R_**s**_ (Ω)**	**CPE1_**film**_ (μFcm^**-2**^)**	**n_**film**_**	**R_**film**_ (kΩcm^**2**^)**	**CPE2_**dl**_ (μFcm^**-2**^)**	***n*2**	**R_**ct**_ (kΩcm^**2**^)**
Cu18Zn	1	−59	614 (8)	18 (30)	0.87 (7)	2 (16)	88 (5)	0.7 (3)	37 (9)
	3	−44	653 (8)	10 (20)	0.93 (7)	5 (23)	59 (6)	0.7 (12)	87 (20)
	16	−48	569 (1)	8 (15)	0.94 (3)	4 (21)	36 (3)	0.7 (2)	95 (5)
Cu28Zn	1	−69	603 (6)	22 (16)	0.83 (2)	4 (21)	71 (19)	0.7 (2)	31 (20)
	3	−49	683 (3)	10 (17)	0.92 (3)	5 (20)	47 (12)	0.7 (1)	12 (14)
	16	−63	514 (1)	11 (15)	0.89 (2)	2 (25)	54 (2)	0.6 (2)	70 (5)
Cu37Zn	1	−83	652 (6)	21 (33)	0.88 (5)	2 (27)	90 (14)	0.6 (3)	32 (12)
	3	−82	565 (10)	18 (18)	0.86 (7)	3 (30)	69 (16)	0.6 (5)	40 (27)
	16	−76	608 (1)	12 (20)	0.96 (3)	1 (14)	86 (2)	0.7 (2)	17 (3)
Cu35Zn1Pb	1	−79	618 (1)	31 (25)	0.84 (7)	3 (30)	91 (17)	0.6 (12)	26 (25)
	3	−66	661 (4)	18 (30)	0.83 (11)	3 (22)	53 (3)	0.7 (5)	56 (30)
	16	−61	651 (5)	14 (20)	0.91 (11)	3 (18)	53 (6)	0.7 (5)	78 (20)
Cu38Zn2Pb	1	−86	623 (10)	53 (6)	0.80 (3)	6 (16)	90 (6)	0.6 (12)	23 (20)
	3	−68	656 (6)	17 (25)	0.91 (8)	4 (23)	58 (13)	0.6 (5)	49 (32)
	16	−75	629 (1)	11 (9)	0.91 (1)	3 (15)	47 (1)	0.6 (2)	91 (4)

**Table 2:** Calculated fitting parameters for brass alloys after 1, 3, and 16 h of immersion in artificial saliva solution.

	**Time (h)**	**OCP (mV)**	**R (Ω)**	**CPE1_**film**_ (μFcm^**-2**^)**	**n_**film**_**	**R_**film**_ (kΩcm^**2**^)**	**CPE2_**dl**_ (μFcm^**-2**^)**	**n_**dl**_**	**R_**ct**_ (kΩcm^**2**^)**
Cu18Zn	1 3	−348 (1) −330 (2)	170 (11) 139 (14)	– 24 (12)	– 0.51 (1)	– 22 (37)	109 (2) 22 (9)	0.81 (1) 0.82 (2)	2 (1) 3 (7)
	16	−159 (20)	139 (16)	93 (26)	0.67 (20)	17 (20)	23 (4)	0.83 (6)	7 (30)
Cu28Zn	1 3	−347 (1) −314 (3)	164 (7) 160 (1)	– 23 (11)	– 0.51 (3)	– 36 (20)	103 (1) 24 (20)	0.82 (1) 0.83 (2)	2 (2) 3 (13)
	16	−230 (3)	160 (2)	11 (38)	0.55 (5)	371 (5)	2 (40)	0.86 (7)	1 (37)
Cu37Zn	1 3	−338 (3) −279 (4)	150 (1) 176 (8)	– 15 (17)	– 0.51 (3)	– 72 (12)	122 (14) 25 (24)	0.77 (1) 0.80 (2)	5 (4) 3 (22)
	16	−320 (7)	159 (2)	21 (5)	0.43 (5)	77 (13)	5 (5)	0.80 (1)	2 (6)
Cu35Zn1Pb	1 3	−344 (1) −277 (12)	158 (4) 164 (4)	– 2 (12)	– 0.52 (3)	– 77 (30)	81 (19) 34 (15)	0.81 (2) 0.77 (3)	4 (16) 3 (19)
	16	−187 (19)	198 (2)	12 (30)	0.50 (3)	427 (18)	2 (33)	0.85 (5)	2 (24)
Cu38Zn2Pb	1 3	−341 (1) −304 (2)	154 (6) 208 (36)	– 10 (32)	– 0.51 (3)	– 44 (18)	115 (2) 34 (22)	0.78 (1) 0.77 (2)	4 (2) 4 (23)
	16	−167 (3)	128 (1)	8 (1)	0.49 (1)	743 (1)	1 (4)	0.86 (3)	1 (10)

*The standard deviation in percentage of the value is obtained from three replica measurements and is reported in parentheses*.

**Figure 3:**
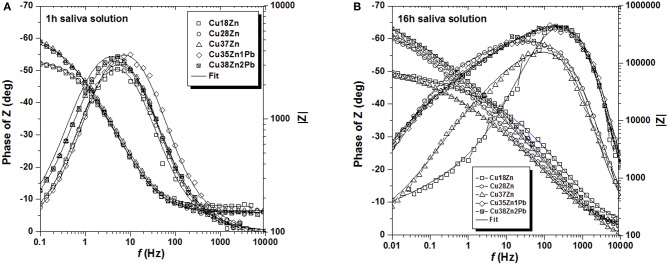
Bode plots of all the brass alloys in contact with the artificial saliva solution for 1 h **(A)** and for 16 h **(B)**.

The impedance spectra of all brass alloys after 1 and 16 h immersion in the artificial saliva solution are shown in Figure 3. In this solution the alloys with lower Zn content clearly show lower impedance values both after 1 and 16 h immersion. The impedance at low frequency is increasing with time and in the phase angle the time constants are more broadly distributed after 16 h of immersion (Figure 3B). Also in saliva solution the Nyquist plots (Figures S.3, S.4) showed only one depressed semicircle. The polarization resistance Rp (extrapolation at the low frequency end) in artificial saliva solutions is much higher than in the phosphate buffer solution.

**Figure 4:**
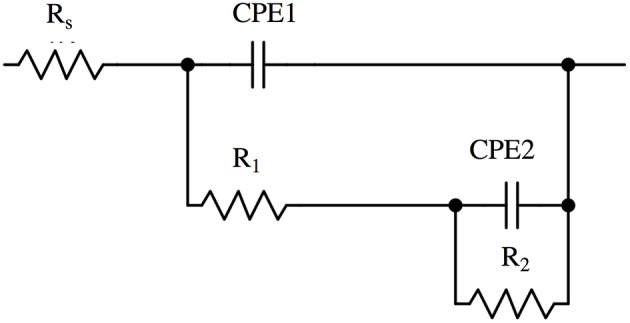
Equivalent circuit used to analyse the impedance spectra of brass alloys in phosphate buffer solution and in artificial saliva solution.

The Bode plots (Figures 2, 3) show two more or less pronounced maxima in the phase angle, indicating two time constants. The EIS data were thus fitted using the equivalent circuit shown in Figure 4, consisting of a solution or ohmic resistance Rs, in series with two time constants, R1/CPE1 and R2/CPE2. The first one, R1/CPE1, was associated to the resistive and capacitive effects of the surface film formed after contact with the solution: R1 can be interpreted as the film resistance, R_film_ (blocking ionic or electronic transport) and the CPE1 as the surface film capacitance, CPE_film_. In the second time constant, R2 could be related to the charge transfer resistance R_ct_ and the related CPE2 describe the capacitance of the double layer, CPE_dl_. The results are summarized in Table 1 (buffer solution) and in Table 2 (artificial saliva solution). As the parameters given are the result of a non-linear curve fitting process, the accuracy of the fit or better the errors in the individual parameters are important. It has been found that the error is usually in the range of a few to 10%.

The results for all brass alloys immersed in *buffer solution pH 7* (Table 1) show that the film resistance R_film_ is very low. It is in the same order for all the brass alloys studied, thus does not depend on the zinc content, and remains nearly constant with the immersion time. This suggests the presence of a thin and non-protective surface film; indeed, the SEM analyses of the surface (Figure 1) do not show a surface film. The charge transfer resistance, R_ct_, instead, in the case of the buffer solution is much higher than the film resistance, R_film_. R_ct_ is similar for the different brass alloys and increasing with time for all alloys. This would indicate that the corrosion reaction in the system brass alloys/buffer solution is charge transfer controlled.

The results obtained by the EIS analysis carried out in *artificial saliva solution*, listed in Table 2 show that the film resistance R_film_ is much higher than the charge transfer resistance R_ct_. The film resistance increases with increasing zinc content in the brass alloys studied. This suggests the presence of an increasingly thick and protective surface film that is forming on the brass surface as is documented by the SEM analysis (Figure 1). The charge transfer resistance, R_ct_, instead, in the case of the artificial saliva solution is much lower than the film resistance, R_film_. R_ct_ is slightly increasing with the zinc content of the different brass alloys and remains nearly constant with the immersion time. This would indicate, that the corrosion reaction in the system brass alloys/artificial saliva solution is controlled by the film resistance.

### XPS Results

The surface chemical state and composition of the brass samples after the exposure to the two neutral solutions was investigated by XPS/XAES surface analysis. Here, results of the Cu37Zn alloy are presented in detail. The survey spectra of Cu37Zn after contact with the pH 7 buffer solution and with the artificial saliva solution are shown in the supporting information together with the XPS/XAES spectra of the other alloys (Figures S.5–S.16). The surface composition of the alloys after mechanical polishing has been published in a previous work (Cocco et al., 2016b). Curve fitting of the spectra was performed using parameters obtained in reference materials as described elsewhere (Cocco et al., 2016b). In particular the differences in KEs and area ratios of the different components ascribed to the different chemical states in XAES signals were constrained and equal to those determined on the reference materials (Cocco et al., 2016b).

### Buffer Solution

Survey spectra of the brass samples following the contact with phosphate buffer solution allow one to identify the presence of copper, zinc and lead (in samples Cu35Zn1Pb and Cu38Zn2Pb), together with oxygen, phosphorus and, in some samples, small amounts of sodium. Carbon is also present due to the presence of the organic contamination layer, which is always detected on the surface of samples exposed to solutions and to the atmosphere. The survey spectra of Cu37Zn brass are provided in Figure S.5.

**Figure 5:**
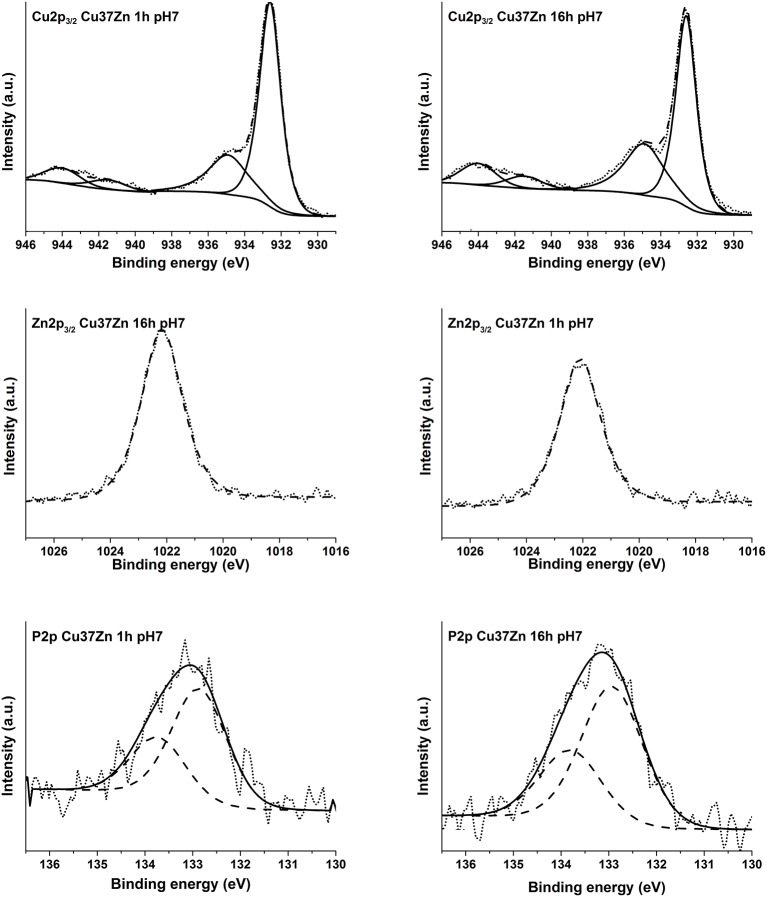
High-resolution spectra of Cu 2p_3/2_, Zn 2p_3/2_, and P 2p for the alloy Cu37Zn after 1, and 16 h of contact with the phosphate buffer solution.

The high-resolution spectra of the signals of Cu2p, CuLMM, Zn2p, ZnLMM, and P2p of the brass alloys were acquired after 1, 3, and 16 h of immersion in the phosphate buffer solution. Examples of the photoelectron spectra of Cu, Zn, and P for the CuZn37 alloy in contact the solution for 1 and 16 h are shown in Figure 5. The binding energy (BE) of the photoelectron lines, the kinetic energy (KE) of the Auger lines and the quantitative composition are given in Table 3.

**Table 3:** Average binding energy (BE) of the most intense photoelectron peaks, kinetic energy of Auger peaks and quantitative composition (at %) of the main elements detected on Cu37Zn after exposure to the phosphate buffer solution.

**CuZn37**	**1 h pH7**		**3 h pH7**		**16 h pH7**	
	**BE (eV)**	**at %**	**BE (eV)**	**at %**	**BE (eV)**	**at %**
Cu 2p_3/2_ Cu (0)	932.5 (0.1)	5 (2)	932.6 (0.1)	1.7 (0.8)	932.6 (0.1)	0.9 (0.4)
Cu 2p_3/2_ Cu (I)	932.5 (0.1)	7 (2)	932.6 (0.1)	12 (3)	932.6 (0.1)	12 (1)
Cu 2p_3/2_ Cu (II)	934.9 (0.1)	5 (2)	934.9 (0.1)	5 (2)	934.9 (0.1)	6 (2)
Sat 1	944.1 (0.1)		944.0 (0.1)		944.0 (0.1)	
Sat 2	941.8 (0.3)		941.5 (0.1)		941.5 (0.1)	
O 1s	530.7 (0.1)	59 (7)	530.6 (0.1)	64 (6)	530.7 (0.1)	64 (7)
	531.7 (0.1)		531.6 (0.1)		531.7 (0.1)	
	533.2 (0.2)		532.5 (0.2)		533.1 (0.2)	
P 2p _3/2_	133.0 (0.2)	12 (3)	132.8 (0.2)	10 (2)	133.0 (0.1)	9 (3)
Zn 2p_3/2_ Zn (0)	1022.1 (0.1)	3 (1)	1022.1 (0.1)	1.7 (0.2)	1022.2 (0.1)	1.4 (0.3)
Zn 2p_3/2_ Zn (II)	1022.1 (0.1)	9 (2)	1022.1 (0.1)	5 (1)	1022.2 (0.1)	3.2 (0.6)
	**KE (eV)**		**KE (eV)**		**KE (eV)**	
Cu L_3_M_4, 5_M_4, 5_ met	918.8 (0.2)		918.8 (0.1)		918.9 (0.2)	
Cu L_3_M_4, 5_M_4, 5_ Ox	916.8 (0.1)		916.9 (0.1)		916.9 (0.2)	
Zn L_3_M_4, 5_M_4, 5_ met	992.3 (0.1)		992.4 (0.1)		992.3 (0.1)	
Zn L_3_M_4, 5_M_4, 5_ Ox	987.4 (0.2)		987.7 (0.2)		987.5 (0.1)	
**Thickness of the layers and composition of surface layer and of the interface bulk/surface layer**						
	**1h pH 7**		**3h pH 7**		**16h pH 7**	
l_c_	1.7 (0.3) nm		1.9 (0.2) nm		2.1 (0.1) nm	
t	0.9 (0.1) nm		1.05 (0.03) nm		0.7 (0.1) nm	
Surface layer	Cu ox = 64 (22)% Zn ox = 36 (22)%		Cu ox = 75 (10)% Zn ox = 25 (10)%		Cu ox = 82 (2)% Zn ox 18 (2)%	
Surface/bulk interface	Cu met = 47 (4)% Zn met = 53 (4)%		Cu met = 47 (9)% Zn met = 53 (9)%		Cu met = 30 (7)% Zn met = 70 (7)%	

*The thicknesses of the contamination layer (lc) and of the surface layer (t), together with the composition of the surface layer and of the bulk/surface layer interface, estimated by the three-layer model (Rossi and Elsener, 1992; Cocco et al., 2016b) are provided. Standard deviations are given in parentheses*.

The detailed Cu 2p_3/2_ signals for all exposure times to the pH 7.4 solution showed two main signals for both short and long contact times: one signal located at BE 932.6 eV related to the presence of Cu (0) and Cu_2_O (Cocco et al., 2016b) and the other signal at about BE 934.9 eV due to the presence of Cu (II) together with the typical satellite structure at the high BE side (944.1 eV Sat 1, 941.5 eV Sat 2) probably due to Cu(OH)_2_ (Biesinger, 2017).

In order to ascertain the presence of Cu (0) following the contact with the buffer solution, the curve fitting approach proposed in Cocco et al. (2016b) for Cu L_3_M_45_M_45_ peaks was applied and the components of the multiplet associated to the metal and to the oxidized copper (^1^G) were found at about KE 918.8 and 916.8 eV, respectively. This suggests the simultaneous presence of Cu (0), Cu (I) and Cu (II). The relative amount of the three different copper species, calculated following (Cocco et al., 2016b), is reported in Table 3. Cu L_3_M_45_M_45_ spectra are shown in the supporting information (Figures S.6–S.16).

The Zn 2p_3/2_ photoelectron signal showed a single peak at a BE of about 1022.1 eV. (Figure 5) As in the case of Cu L_3_M_45_M_45_ also the Zn L_3_M_45_M_45_ Auger signal exhibited both the metallic and the oxidized Zn (II) components; their KEs are 991.3 and 987.5 eV, respectively. The former component is assigned to Zn (0) and the latter might be assigned to zinc oxide and hydrogen phosphate according to the literature (Onyiriuka, 1993; Cocco et al., 2016b). The P 2p_3/2_ signal was found at a BE of about 132.8–133.0 eV: an in depth discussion on P chemical state is provided in the following section (Onyiriuka, 1993).

The binding energies of the photoelectron peaks as well as the kinetic energies of the XAES signals are the same for all the alloys studied as shown in Table 3. This indicates that irrespective of the zinc content of the alloy the same chemical compounds are present on the surface after exposure to the phosphate buffer solution.

The amount of oxidized zinc and copper species increases upon contact time. The application of a three layer model to XPS areas of carbon, oxygen, metallic copper and zinc and oxidized copper and zinc allows us to estimate the thickness of the organic contamination layer (l_c_), of the surface layer containing Cu (I), Cu (II), and Zn (II) species as oxides, hydroxides and hydrogen phosphates (t), and the composition of the surface layer and of the interface bulk/surface layer. The surface layer is richer in copper, mainly as Cu_2_O and Cu(OH)_2_, than in zinc, as oxide and/or hydrogen phosphate. On the contrary, the bulk/surface layer interface is enriched in zinc in comparison with the composition of the interface in the mechanically polished Cu37Zn. In this case the interface was found to be close to the nominal composition (63% copper, 37% zinc) (Cocco et al., 2016b). Zn content of the interface increases upon immersion time.

### Artificial Saliva Solution

**Figure 6:**
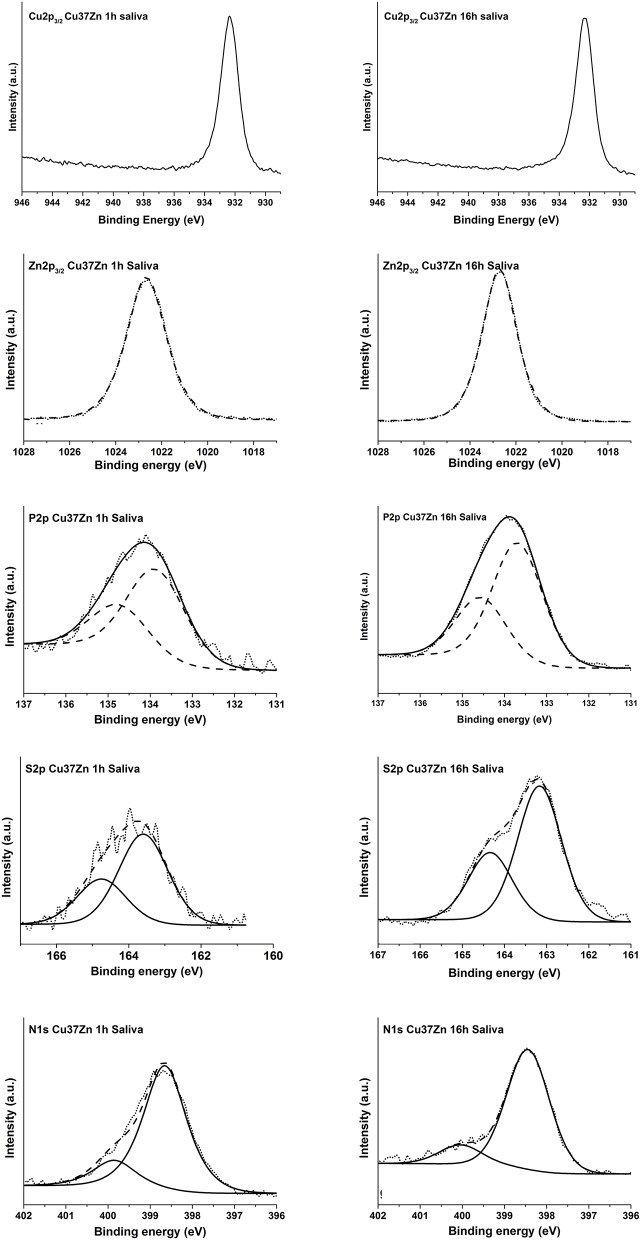
High resolution spectra of Cu 2p_3/2_, Zn 2p_3/2_, P 2p, S 2p, and N 1s of the Cu37Zn sample after 1, 3, and 16 h of contact with the saliva solution.

The high-resolution spectra of the signals Cu 2p, Cu L_3_M_4, 5_M_4, 5_, Zn 2p, Zn L_3_M_4, 5_M_4, 5_, P 2p, S 2p, and N 1s of the brass alloys were acquired after 1, 3, and 16 h of immersion in the saliva solution. As an example the spectra of the CuZn37 alloy are shown in Figure 6. The binding energy (BE) of the photoelectron peaks and the kinetic energy (KE) of the XAES signals together with the atomic concentration calculated excluding the carbon intensity are provided in Table 4. The results related to the other alloys are comparable with those obtained for the here-presented sample and are shown in Tables S.1–S.3.

**Table 4:** Average binding energy (BE) of the most intense photoelectron peaks, kinetic energy of the Auger peaks and quantitative composition (at %) of the main elements detected on Cu37Zn after contact with the saliva solution.

**CuZn37**	**1 h**		**3 h**		**16 h**	
	**BE (eV)**	**at %**	**BE (eV)**	**at %**	**BE (eV)**	**at %**
Cu 2p	932.6 (0.1)	13 (1)	932.5 (0.1)	13 (4)	932.5 (0.1)	6 (1)
N 1s	398.6 (0.1)	10 (2)	398.5 (0.1)	25 (16)	398.5 (0.1)	5 (1)
N 1s	399.9 (0.2)		400.0 (0.1)		400.1 (0.1)	
O 1s	531.1 (0.2)	40 (4)	532.1 (0.2)	29 (3)	532.4 (0.1)	50 (1)
O 1s	532.3 (0.2)		532.5 (0.2)		533.6 (0.1)	
O 1s	533.3 (0.2)		533.0 (0.1)		531.7 (0.1)	
P 2p _3/2_	134.2 (0.2)	7(1)	134.3 (0.2)	6 (1)	133.8 (0.1)	13 (2)
S 2p SCN	163.3 (0.2)	11 (1)	163.3 (0.1)	15 (7)	163.2 (0.1)	6 (1)
Zn 2p	1022.9 (0.2)	18 (1)	1022.8 (0.1)	12 (1)	1022.7 (0.1)	20 (3)
	**KE (eV)**		**KE (eV)**		**KE (eV)**	
Cu L_3_M_4, 5_M_4, 5_	915.7 (0.1)		915.5 (0.1)		915.5 (0.1)	
Zn L_3_M_4, 5_M_4, 5_	986.3 (0.1)		986.2 (0.2)		986.3 (0.1)	

*Standard deviations are given in parentheses*.

The Cu 2p_3/2_ signal showed a single peak at 932.6 eV for both short and long contact times and for all the analyzed samples. The Cu L_3_M_45_M_45_ Auger signals were composed of five peaks associated to different final states of oxidized copper (Cocco et al., 2016b), here only the envelope of the XAES signal is shown. No metallic component has been detected in the CuLMM Auger spectra even after only 1 h of exposure; hence the thickness of the film formed was greater than the sampling depth of the XPS technique. The kinetic energy of the main component of the Cu LMM signals was found at about 915.7 eV for all the alloys. The Cu L_3_M_45_M_45_ Auger peak overlaps with the Zn L_2_M_45_M_45_ (black line in Figure 6) on the high KE side of the main peak. After 16 h of immersion this superposition is the highest, this could be explained as a result of the increasing zinc content in the film formed on the surface of the alloy.

The KE values of the main Cu L_3_M_45_M_45_ peak could be assigned to the presence of copper thiocyanate, CuSCN according to Cocco et al. (2016b). Moreover, this assignment was also confirmed by the presence of N 1s and S 2p and by their BE values. The N 1s spectra showed two components: (1) the most intense peak at about 398.5 eV might be due to nitrogen in SCN^−^ (Pattanasattayavong et al., 2013; Aldakov et al., 2014)^2^; (2) the less intense component at about 399.9 eV is probably due to residual urea from saliva formulation^2^. The S 2p_3/2_ signal was found at 163.3 eV and its BE does not change upon exposure time. The position of the signal might be ascribed to a sulfur atom in ^−^S–CN^2^.

The Zn 2p_3/2_ spectra (Figure 6) showed a single peak at about 1022.8 eV for each exposure time and for all the alloys. The related Zn L_3_M_45_M_45_ Auger signals exhibited a complex shape with five components as a result of different final states; the main peak was located at KE 986.3 eV. This could be assigned to the presence of oxidized zinc in the form of zinc orthophosphate according to literature (Crobu et al., 2012; Cocco et al., 2016b). The P 2p showed a well-separated spin-orbit doublet; the energy separation between 2p_3/2_ and 2p_1/2_ is 1.2 eV. The P 2p_3/2_ signal was found at 133.8 eV and it was likely assigned to the presence of phosphate group according to Cocco et al. (2016a).

Unlike the case of the buffer solution where the metallic components are still detectable after 16 h of exposure, no signals from Cu (0) and Zn (0) were revealed (Figure 6), indicating that in saliva solution a thicker film was formed on the brass surface.

## Discussion

In a previous work (Cocco et al., 2016a), based on open circuit potential, DC polarization resistance measurements, and preliminary XPS/XAES surface analysis data, it was hypothesized that the dissolution of the brass alloys exposed to phosphate buffer solution and to artificial saliva solution was controlled by the different surface films formed. Using electrochemical impedance spectroscopy (EIS) and a full set of XPS/XAES surface analysis data, a more detailed insight into the reaction mechanism of the brass alloys with zinc content ranging from 18 to 38% exposed to the two different solutions can be provided and the rate controlling step can be established. This information is important for brass wind instruments where corrosion occurs due to the high internal humidity formed after playing.

### Curve Fitting and Interpretation of the Impedance Spectra

Electrochemical impedance spectroscopy (EIS) is a powerful tool to study the reaction mechanism of corrosion processes as the technique allows differentiating between various partial processes and reaction steps. Today the use of software packages that allow simulating different processes and adapting the calculations to the experimental spectra facilitates greatly their interpretation. However, two points should be considered: first, every element in an equivalent circuit has to have a physical meaning, and second, the errors associated to the individual elements resulting from the curve fitting procedure should be visible.

The EIS spectra in this work (Figures S.1, S.2) showed one broad or two resolved maxima in the phase angle, indicating two time constants related to two different reactions at the surface of the brass alloys exposed to the solutions. The corresponding equivalent circuit (Figure 4) has, in addition to the ohmic resistance R_Ω_, two time constants: R1/CPE1 and R2/CPE2. The constant phase element (CPE) was used instead of a capacitance C to take into account a distributed capacitance due to surface roughness, variation in film thickness etc. resulting in an exponent *n* < 1. Overall there is a good agreement between the experimental and calculated spectra (Figures 2, 3).

Examining the results of the adaption of the equivalent circuit to the experimental data (curve fitting procedure) given in Tables 2, 3 in more detail, it can be noted that the errors associated to the ohmic resistance R_Ω_ are generally lower than 10%. The film resistance R_film_ in the phosphate buffer solutions (Table 2) is very low (in the range of 1–4 kΩ cm^2^), the associated error is between 20 and 50%. The error in the charge transfer resistance R_ct_ is generally below 25%.

### Dissolution Mechanism of Brass Alloys

In this section the dissolution mechanism of the brass alloys in the two solutions is discussed combining the results of electrochemical impedance spectroscopy with the results of the surface analysis.

### Electrochemical Impedance Spectroscopy

The analysis and interpretation of the impedance spectra recorded on the brass alloys after 1, 3, and 16 h of exposure to the phosphate buffer solution and to artificial saliva allowed determining the film resistance R_film_ and the charge transfer resistance, R_ct_. R_ct_ provides information on the rate of the anodic dissolution reaction of the brass alloy whereas the film resistance R_film_ indicates to what extent the actual corrosion rate is limited by the surface film that was formed as a result of the alloy dissolution.

**Figure 7:**
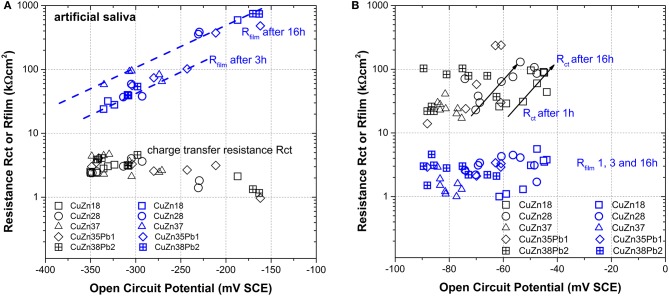
Scatter plot of the film resistance R_film_ and the charge transfer resistance R_ct_ vs. OCP for all alloys and immersion times in **(A)** artificial saliva and in **(B)** phosphate buffer solution.

Plotting both parameters for the artificial saliva solution (Figure 7A) and for the phosphate buffer solution (Figure 7B) as a function of the open circuit potential, a net result is obtained:

∘ In the *artificial saliva solution* R_film_ >> R_ct_, thus the dissolution reaction is controlled by the surface film in agreement with the results of the DC polarization resistance measurements where an anodic control was found (Cocco et al., 2016a). The charge transfer resistance does not vary with the zinc content of the alloy and with immersion time whereas the film resistance increases with time of immersion from 3 to 16 h.
∘ In the *phosphate buffer* solution R_ct_ >> R_film_, thus the dissolution reaction of the alloy is controlled by the charge transfer reaction. The film resistance remains constant at 4 ± 2 kΩcm^2^ independent of the zinc content in the alloy or of the exposure time. The charge transfer resistance increases with time for all alloys from about 20–100 kΩcm^2^, the difference between the alloys is a slight shift in the OCP values with the zinc content—a trend already found in the DC measurements (Cocco et al., 2016a).

In conclusion, in the phosphate buffer solution the dissolution rate is charge transfer controlled, in the artificial saliva the film resistance governs the dissolution rate.

### Surface analysis

The dissolution mechanism of the brass alloys in the two solutions could be well-established based on the EIS results. What has to be discussed further is the link to the results of the XPS/XAES surface analysis, in other words how does the composition of the surface film influence the dissolution rate.

∘ In the *artificial saliva solution* a thick film is formed after 3 and 16 h of immersion (Figure 1) showing a surface morphology with numerous crystallites. No signals from the metal could be detected for all the alloys as it could be seen from the XAES spectra of both Cu LMM and Zn LMM (Figure 4) even after 1 h of immersion the film thickness thus is much higher than the escape depth of the photoelectrons. First results revealed, based on the Wagner chemical state plot (Cocco et al., 2016a) the presence of copper thiocyanate (CuSCN) and zinc phosphate. Quantitative analysis of the surface film in this work indicated 22 ± 3 at % zinc, 14 ± 3 at % phosphorus and about 64 ± 5 at % oxygen, thus the presence of Zn_3_(PO_4_)_2_ is confirmed. The increase of the film resistance with time can be interpreted as an increase in the film thickness or the formation of a more compact film; this is also reflected in the decrease of the film capacitance (Table 2).
∘ In the *phosphate buffer solution* no visible film is formed, the morphology is similar to the mechanically polished surface (Figure 1). For all the brass alloys even after 16 h of immersion the presence of the metallic component of both Cu and Zn was detected in the high-resolution XAES signals of Cu LMM (Figure 5) and in the Zn LMM spectrum (Figure 5) respectively, qualitatively indicating the presence of a surface film in the order of some nano-meters only. This is in agreement with studies on the effect of phosphate ions on copper dissolution and passivation (Drogowska et al., 1992; Antonijevic and Petrovic, 2008; Valcarce and Vázquez, 2010; Deyab et al., 2015). In phosphate solution of pH 8 cyclic voltammetry showed that the phosphate ions were involved in the reaction. The thickness of the anodic film was estimated (based on the reduction charge) to 40–48 A when a film composition of CuO or Cu(OH)_2_ was assumed (Cocco et al., 2016a). In an earlier work of Strehblow and Titze (1980) the surface film of copper exposed to phosphate solution was studied. In addition to Cu_2_O and Cu(OH)_2_ the presence of a copper phosphate compound, most probably Cu_3_(PO_4_)_2_, was indicated as possible explanation of the traces of phosphorus revealed by XPS.

**Figure 8:**
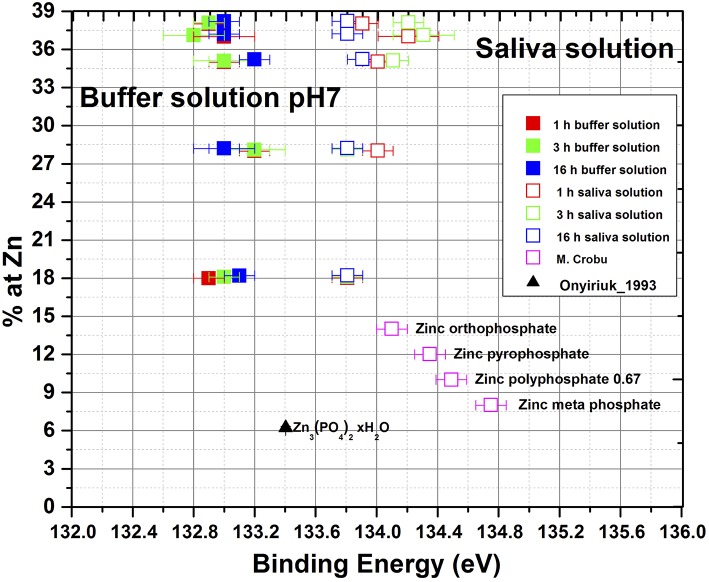
P2p_3/2_ binding energy of surface films formed on brass alloys in artificial saliva and in phosphate buffer solution together with phosphate compounds.

In the context of the nature of these surface films containing phosphates the detailed analysis of the P2p_3/2_ binding energy (given in Table 4) is interesting. As is shown in Figure 8, the P2p_3/2_ BE in artificial saliva is found at 133.8 ± 0.2 eV with a slight trend toward high values at higher zinc contents. These binding energy values well-agree with previously measured compounds of zinc orthophosphate glass (Crobu et al., 2012). The presence of Zn_3_(PO_4_)_2_ was determined on the brass samples immersed in artificial saliva with the help of the chemical state plot (Cocco et al., 2016a). On brass samples immersed in phosphate buffer solutions the binding energy of P2p_3/2_ is found at 133.0 ± 0.2 eV for all alloys and immersion times (Figure 8), clearly lower than in saliva solution. Thus, the presence of pure zinc phosphate can be ruled out. The binding energy of pure Cu_3_(PO_4_)_2_ on the other hand was found at 133.9 ± 0.15 eV (Biesinger, 2017) and the presence of pure copper phosphate can also be excluded. A possible explanation of the low binding energy of P2p_3/2_ signal (BE 132.9 ± 0.1 eV, Figure 8) might be that in the thin surface film mainly composed of Cu_2_O, ZnO, and Cu(OH)_2_, the concentration of phosphate is low and the film is hydrated, copper hydrogen phosphate might be present. The binding energy of Na_2_HPO_4_ was reported at 133.0 eV.

### Implications for the Interior Corrosion of Brass Wind Instruments

The motivation for this research project was based on the idea to use period brass musical instruments of the nineteenth and twentieth century for today concerts in the context of historically informed musical practice (Butt, 2002)^1^. Main problem is the corrosion inside the instruments due to the high humidity formed immediately during playing. From the point of view of museum conservators this can—in principle—not be tolerated in the very old, precious instruments, so the corrosion rate and mechanism of corrosion of the brass alloys had to be investigated. In a series of works it could be established using a dedicated electrochemical sensor that the highest corrosion rates of more than 20 μm/year were observed on freshly polished brass surfaces (Elsener et al., 2016a,b). Brass alloys or parts of old instruments covered with an oxide film showed much lower corrosion rates.

In this work freshly polished CuZn37 brass alloys was exposed to artificial saliva solution, a situation that could be encountered when playing brass wind instruments: measurements showed a high relative humidity inside the instruments after short playing time that remained for several days afterwards (Von Steiger et al., 2015). When analyzing the instrument with neutron computer tomography during playing (simulated with a flux of wet air) even condensation of solution was found (Von Steiger et al., 2015)—thus “artificial saliva” might be a severe but realistic environment inside the tuning slides. The possible formation of a protective surface film as reported in this work might contribute to markedly reduce the corrosion attack. In addition a drying treatment of the interior parts of the instrument with a fan was found to reduce the corrosion rate at least by a factor of two. The average corrosion rates measured after drying were lower than 3 μm/year and thus acceptable for playing the precious historic instruments in concerts (Von Steiger et al., 2018).

## Conclusions

This work combining XPS surface analysis and electrochemical impedance spectroscopy in studying brass alloys with 18–37% of zinc in neutral phosphate buffer or saliva solution allows drawing the following conclusions:

Electrochemical impedance spectroscopy (EIS) allowed revealing the dissolution mechanism of the alloys in the two solutions studied:

- In the phosphate buffer solution the dissolution rate is charge transfer controlled, prolonged exposure time up to 16 h had only a minor influence on the corrosion rate.
- In the artificial saliva solution, the dissolution rate of the brass alloys is controlled by the resistance of the protective film formed on the surface, the corrosion rate decreased with prolonged exposure time. The charge transfer resistance was found at about 3 ± 1 kΩcm^2^ for all alloys and exposure times.

Quantitative analysis of the surface films formed on the brass alloys in artificial saliva solution by XPS/XAES spectroscopy indicated 22 ± 3 at % zinc, 14 ± 3 at % phosphorus and about 64 ± 5 at % oxygen, thus the presence of Zn_3_(PO_4_)_2_ indicated in the chemical state plot is confirmed. The increase of the film resistance with time can be interpreted as an increase in the film thickness and/or the formation of a more compact film.

The surface film formed in the phosphate buffer solution on all the brass alloys and even after 16 h of immersion shows a thickness of some nanometres only as the presence of the metallic component of both Cu and Zn was detected in the high-resolution XAES spectra. This can explain the charge transfer controlled dissolution mechanism of the brass alloys in this solution.

## Data Availability Statement

The raw data supporting the conclusions of this article will be made available by the authors, without undue reservation, to any qualified researcher.

## Author Contributions

AR and BE: conceptualization. FC, MF, and CP: XPS/XAES, electrochemical, SEM acquisition and processing, and writing of the original draft. BE, AR, and MF: methodology, supervision, validation, writing, reviewing, and editing.

### Conflict of Interest

The authors declare that the research was conducted in the absence of any commercial or financial relationships that could be construed as a potential conflict of interest.

## Footnotes

^1^Web site of the project “Brass instruments of the nineteenth and early twentieth centuries between long-term conservation and use in historically informed performance practice” Available online at: http://p3.snf.ch/Project-146330.

^2^https://srdata.nist.gov/xps/ (access October 11, 2016).
