# Supplementary material for: Model Protective Films on Cu-Zn Alloys Simulating the Inner Surfaces of Historical Brass Wind Instruments by EIS and XPS
Source: Front Chem. 2020 Apr 15;8:272. doi: 10.3389/fchem.2020.00272 (PMC7174669; doi:10.3389/fchem.2020.00272)
Supplement: Supplementary file 1 [file Data_Sheet_1.docx]

**Supplementary material**

**Model Protective Films on Cu-Zn Alloys Simulating the Inner Surfaces of Historical Brass Wind Instruments by EIS and XPS**

Marzia Fantauzzi^1)^, Bernhard Elsener^1) 2)^, Federica Cocco^1)^, Cristiana Passiu ^3)^, Antonella Rossi^1)^

1. Dipartimento di Scienze Chimiche e Geologiche, Università degli Studi di Cagliari, Campus di Monserrato S.S. 554 – Italy and INSTM, UdR Cagliari – Italy
2. Institute for Building Materials, ETH Zurich, ETH Hönggerberg, CH-8093 Zurich, Switzerland
3. Department of Materials, Laboratory for Surface Science and Technology, ETH Zurich, Vladimir-Prelog-Weg 5, CH-8093 Zurich, Switzerland

* Corresponding author

# Impedadance results


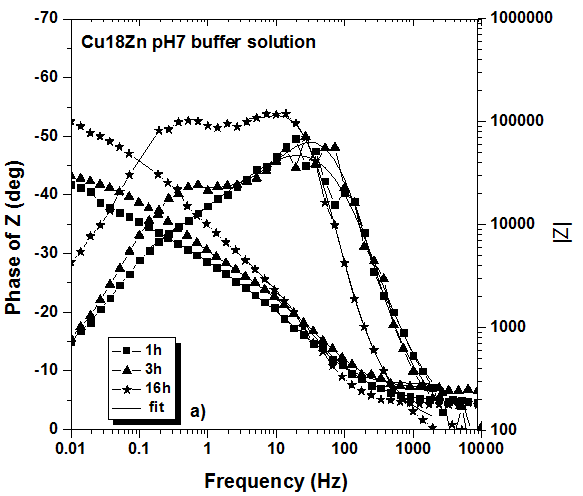

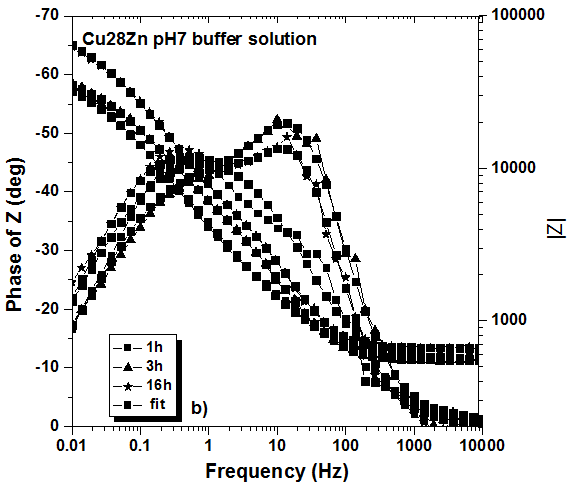

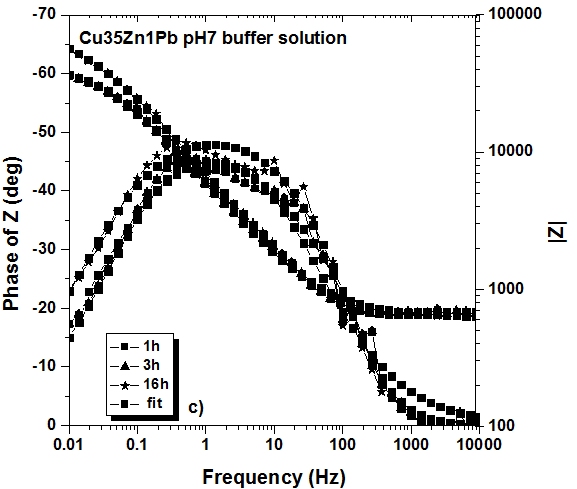


**Figure S.1:** Bode plots of the Cu18Zn (a), Cu28Zn (b) and Cu35Zn1Pb (c) in contact with the pH7 model solution.


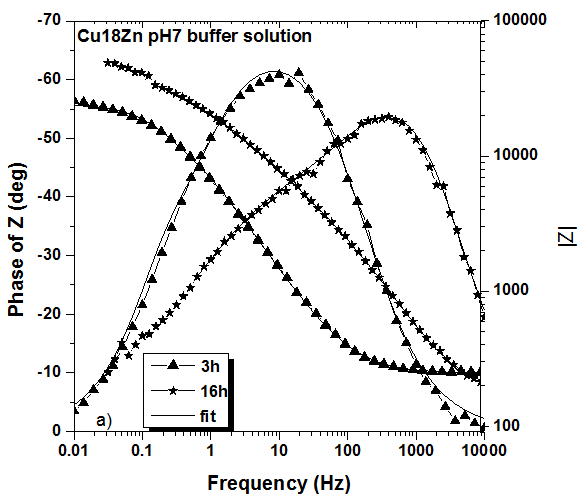

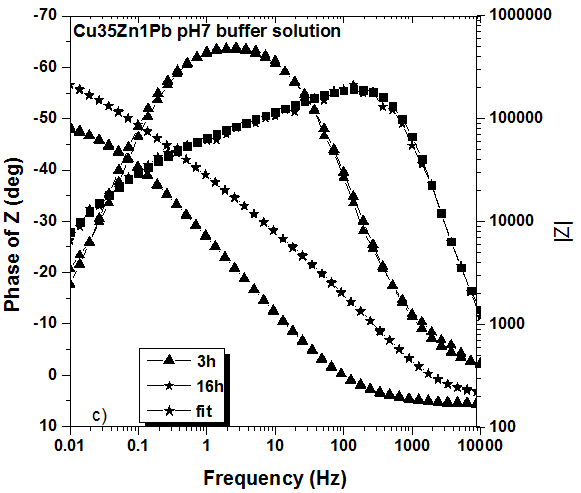

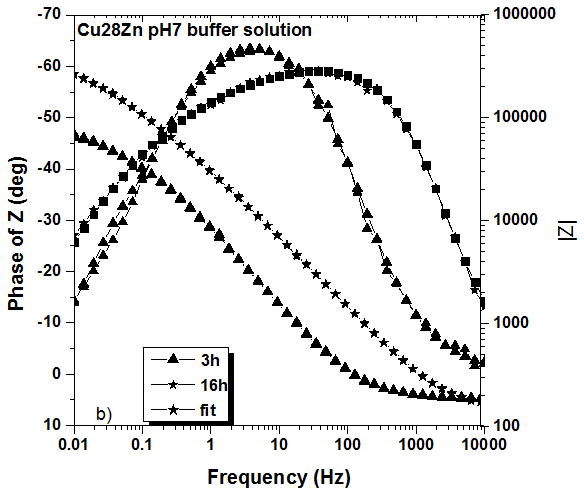


**Figure S.2:** Bode plots of the Cu18Zn (a), Cu28Zn (b), and Cu35Zn1Pb (c) in contact with the artificial saliva solution.


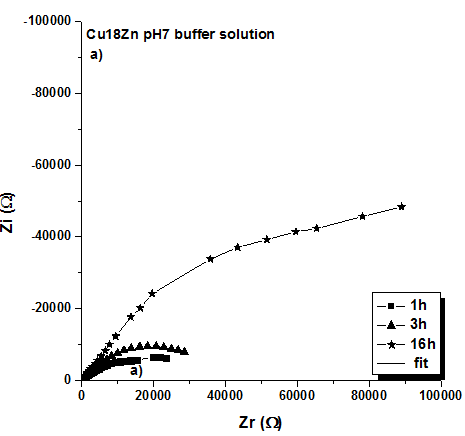

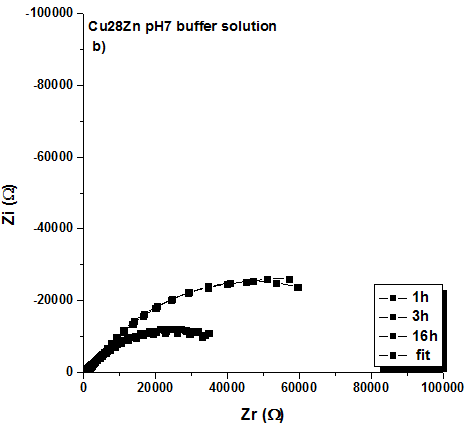

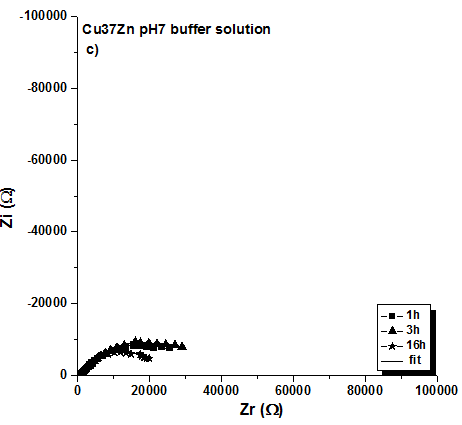

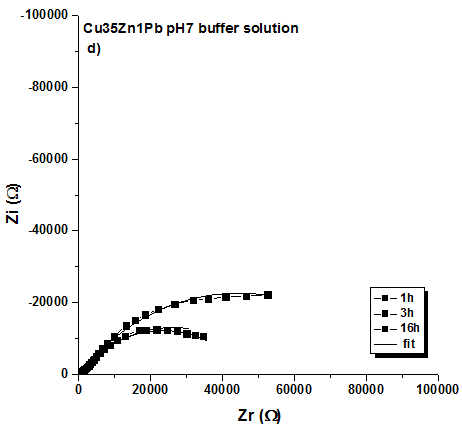

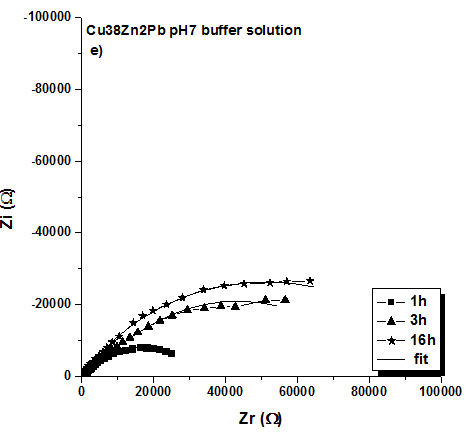


**Figure S.3:** Nyquist plots of the Cu18Zn (a), Cu28Zn (b), Cu37Zn (c), Cu35Zn1Pb (d), and Cu38Zn2Pb (e) in contact with the pH7 model solution. The frequency decreases from the right towards the zero on the x-axis.


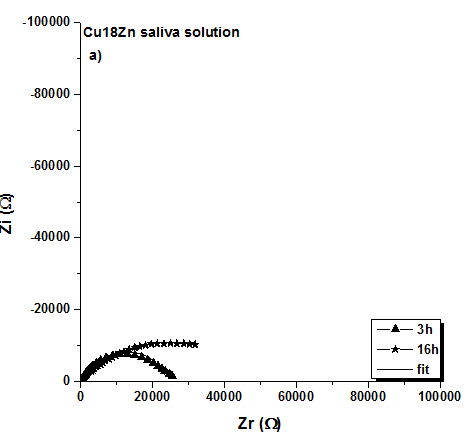

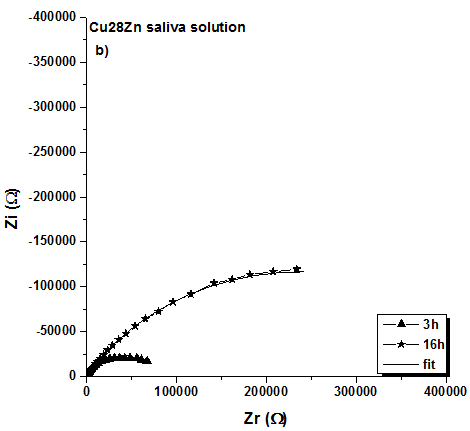

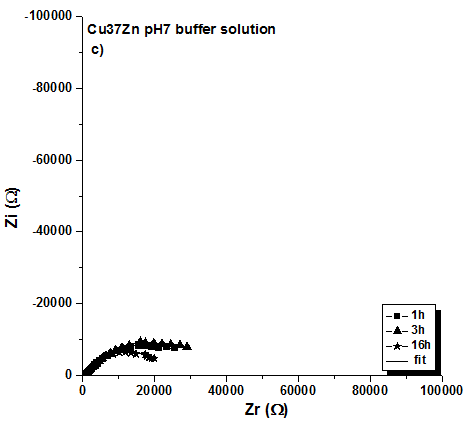

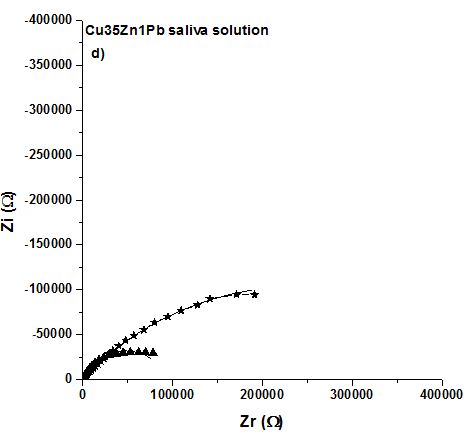

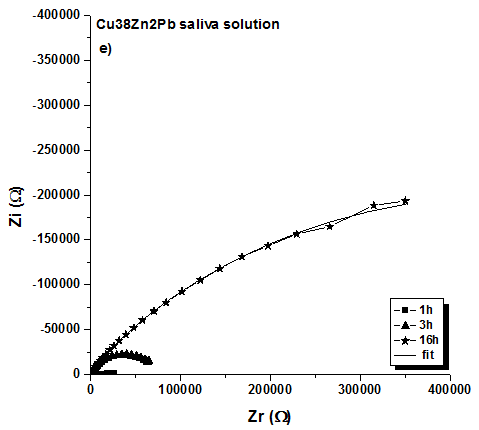


**Figure S.4:** Nyquist plots of the Cu18Zn (a), Cu28Zn (b), Cu37Zn (c), Cu35Zn1Pb (d), and Cu38Zn2Pb (e) in contact with the artificial saliva solution. The frequency decreases from the right towards the zero on the x-axis.

# XPS results

**2.2 Figures**




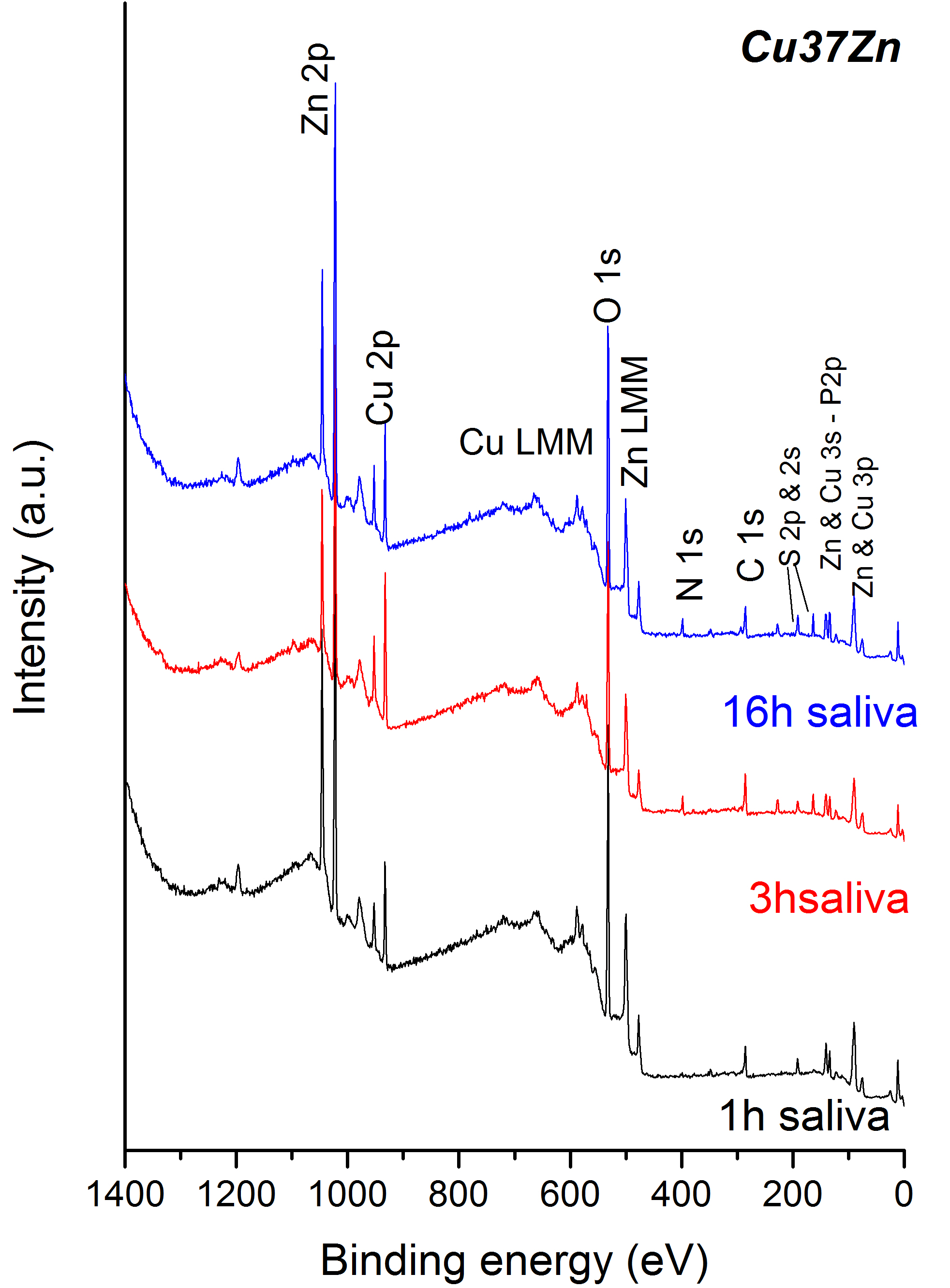


**Figure S.5** Survey spectra of Cu37Zn exposed to buffer solution (left side) and to artificial saliva (right side) for 1h, 3h and 16 h


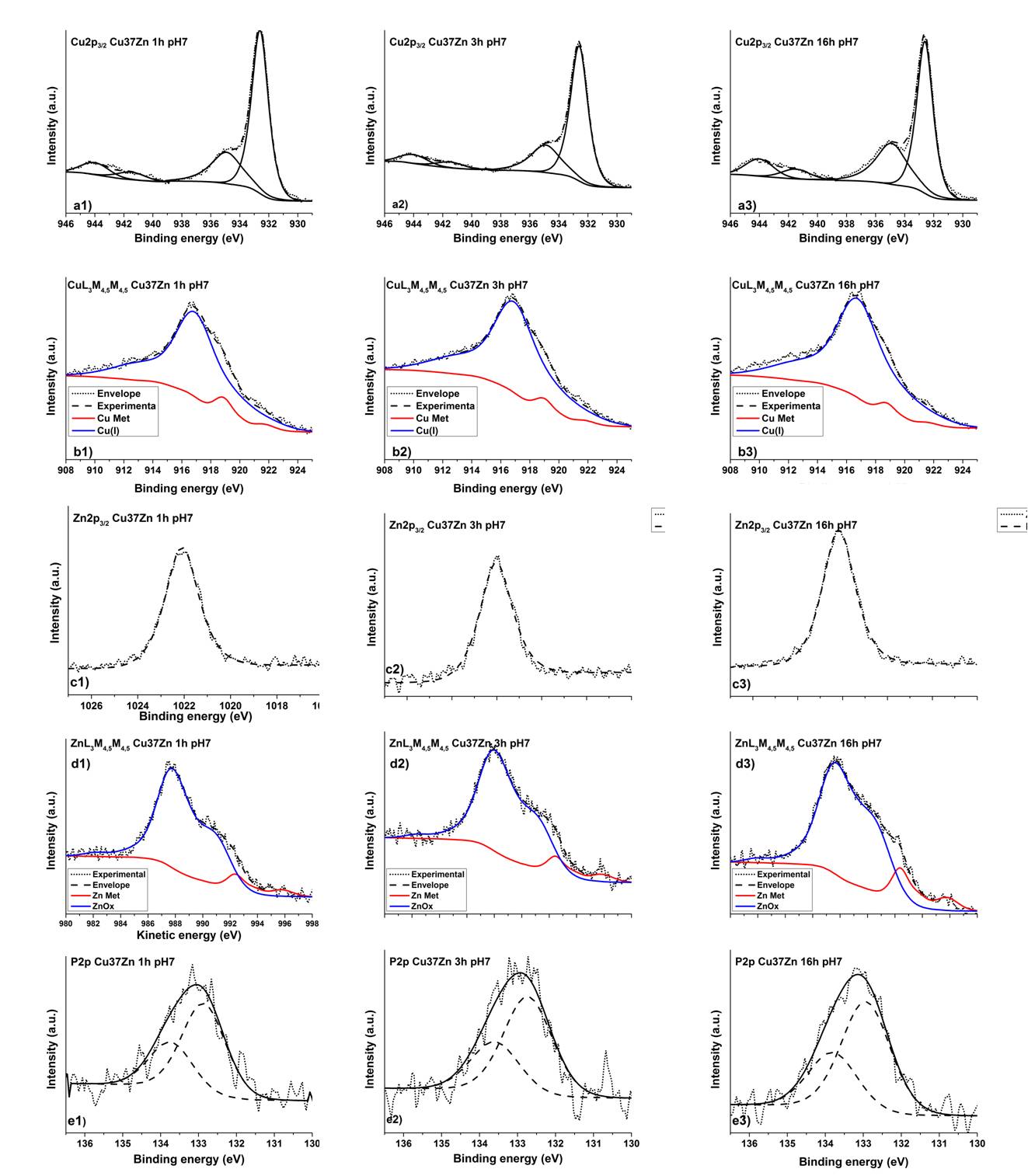


**Figure S.6:** High resolution spectra of Cu 2p_3/2_ (a), Cu L_3_M_45_M_45_ (b), Zn 2p_3/2_ (c), Zn L_3_M_45_M_45_ (f) and P 2p (e) for the alloy Cu37Zn after 1, 3, and 16 hours of contact with the phosphate buffer solution.


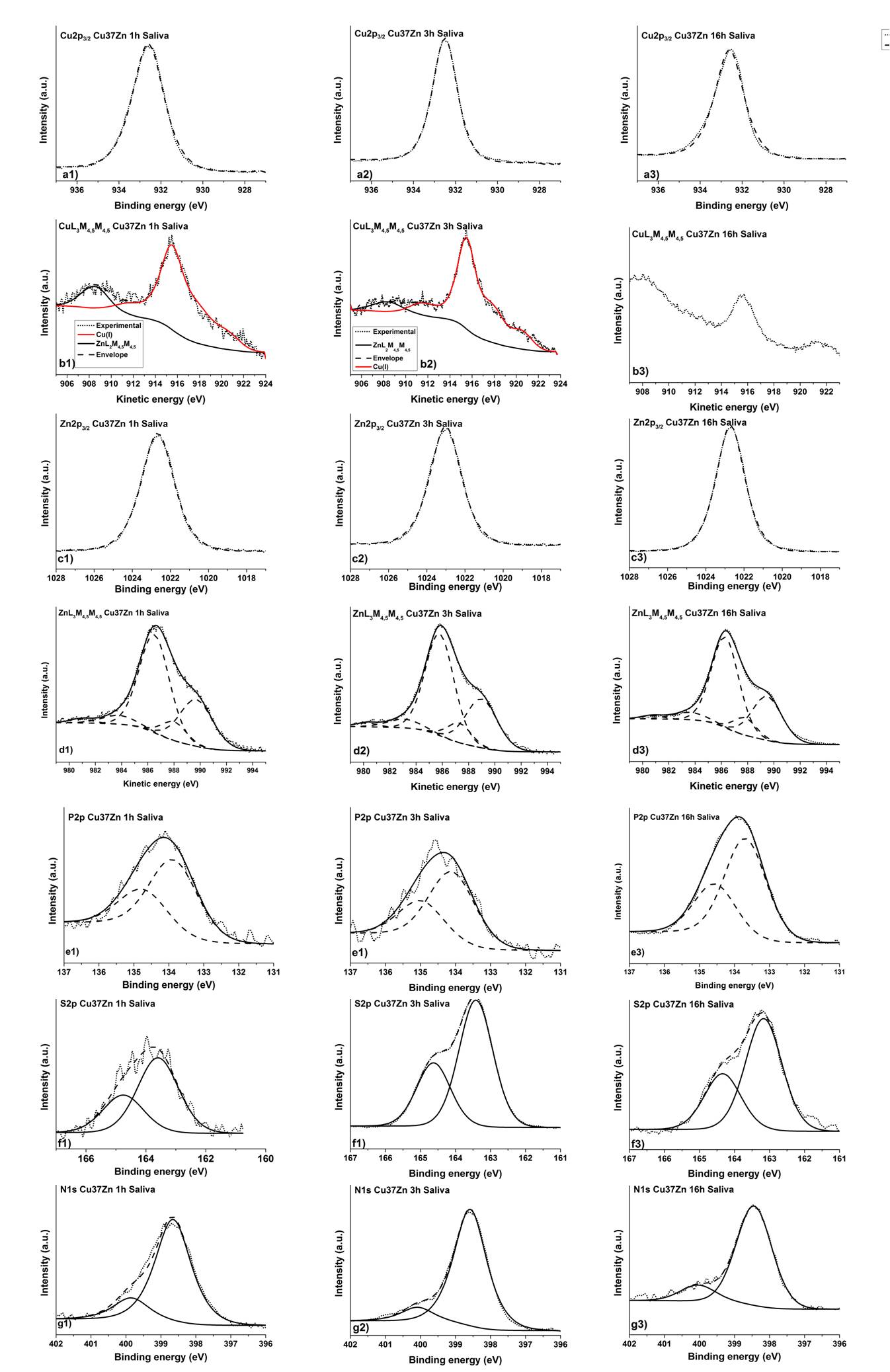


**Figure S.7:** High resolution spectra of Cu 2p_3/2_ (a), Cu L_3_M_45_M_45_ (b), Zn 2p_3/2_ (c), Zn L_3_M_45_M_45_ (d), P 2p (e), S 2p (f) and N 1s (g) of the Cu37Zn sample after 1 (1), 3 (2), and 16 (3) hours of contact with the saliva solution.


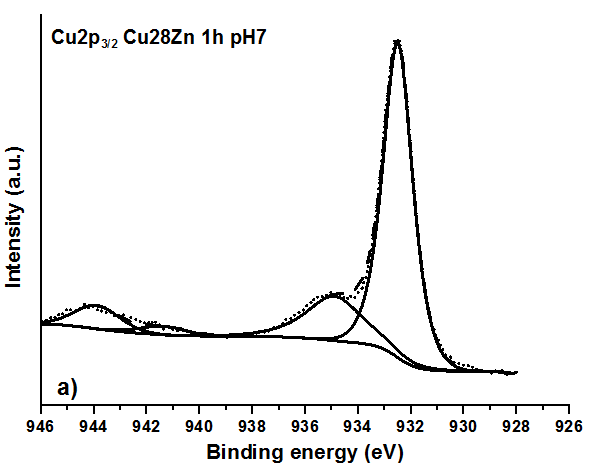

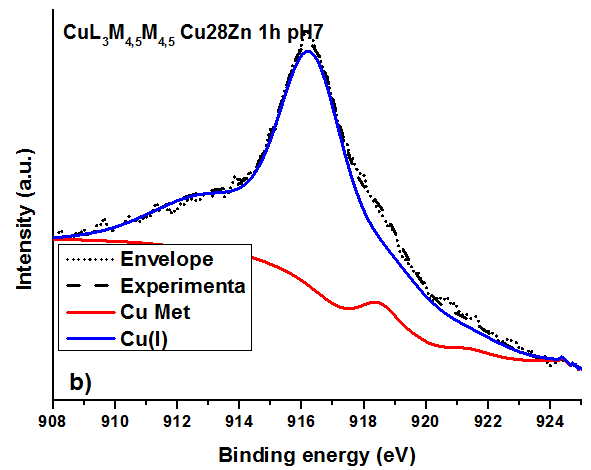

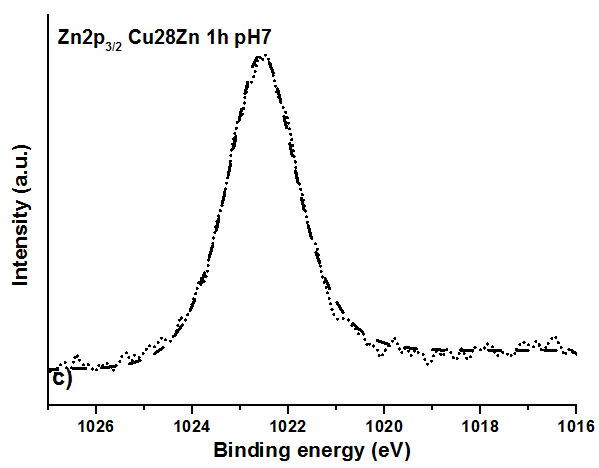

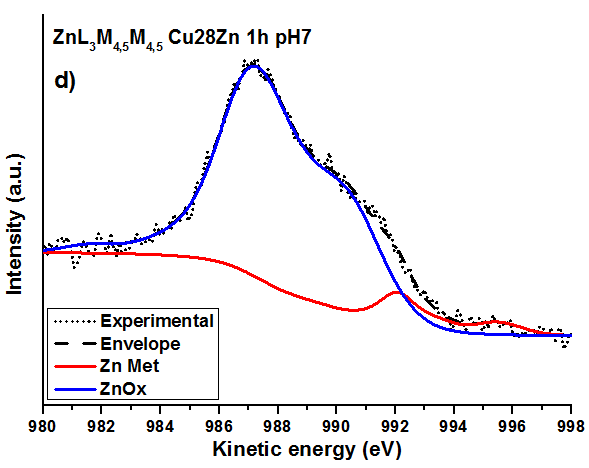

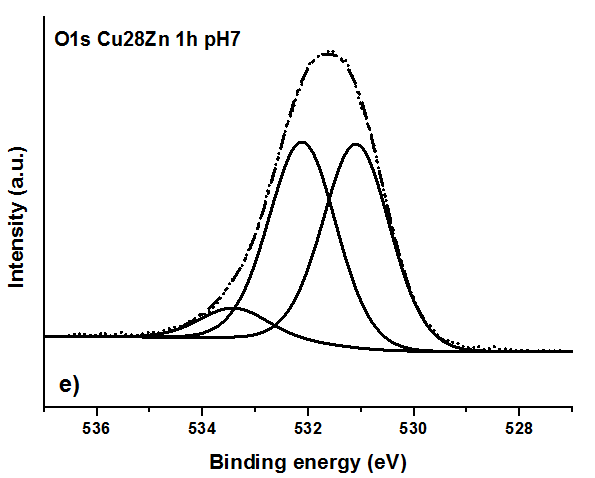

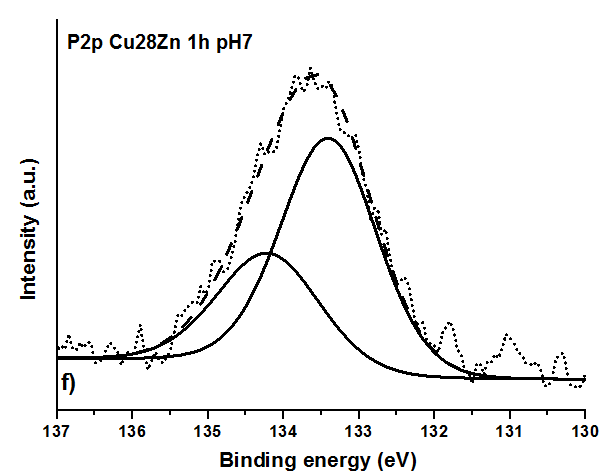


**Figure S.8:** Cu 2p_3/2_ (a), Cu L_3_M_45_M_45_ (b), Zn 2p_3/2_ (c), Zn L_3_M_45_M_45_ (d), O 1s (e), P 2p (f) signals for the Cu28Zn after 1 hour of exposure to the phosphate buffer solution.


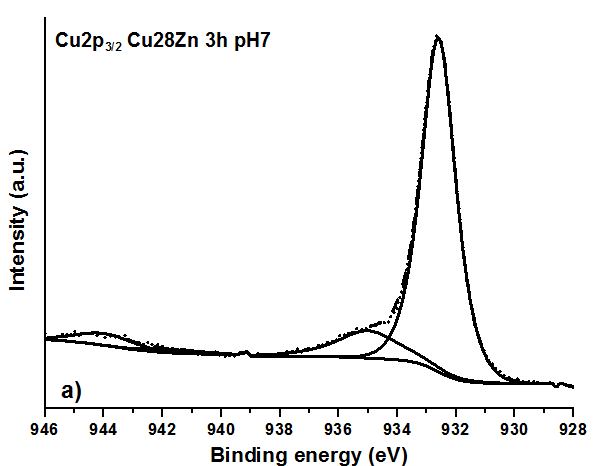

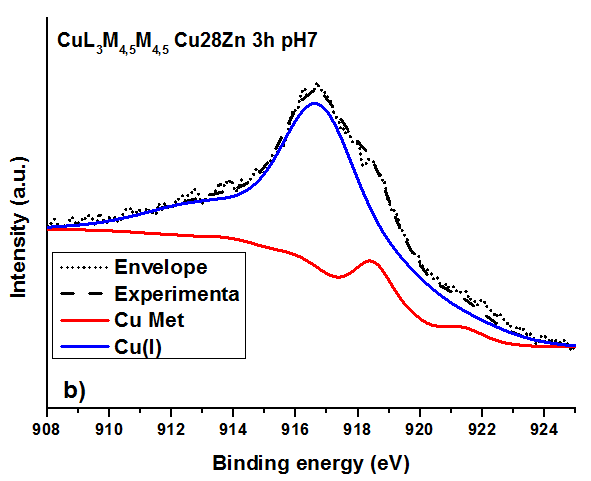

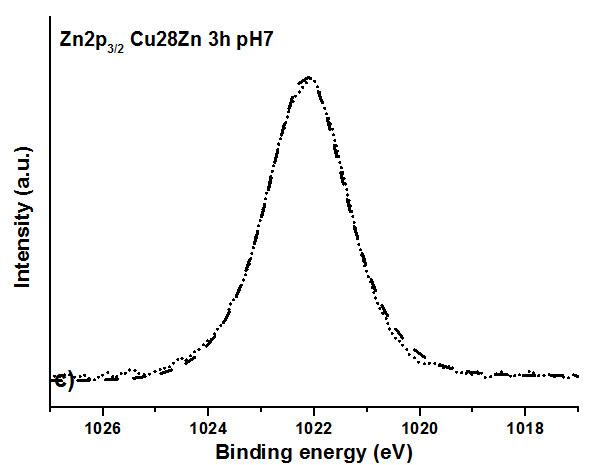

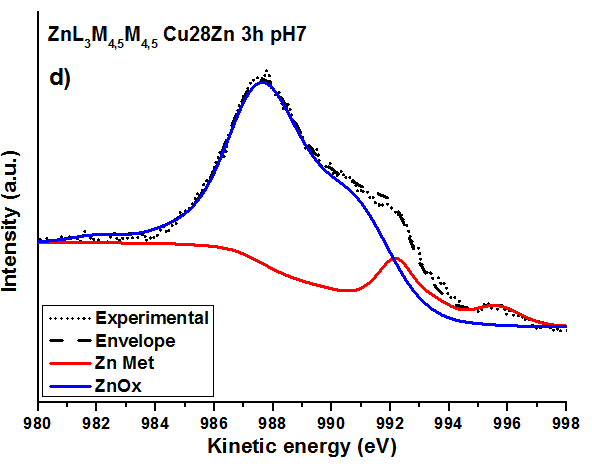

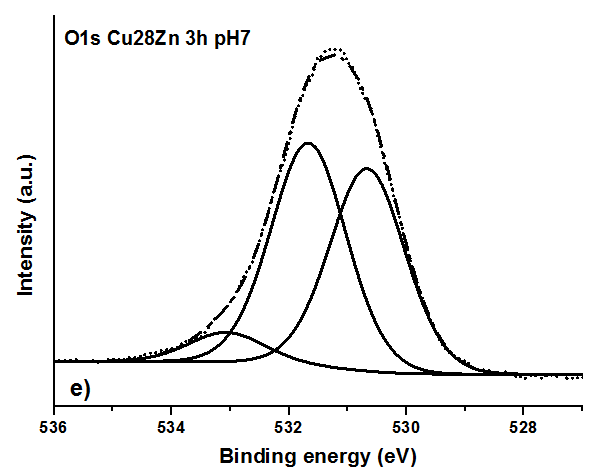

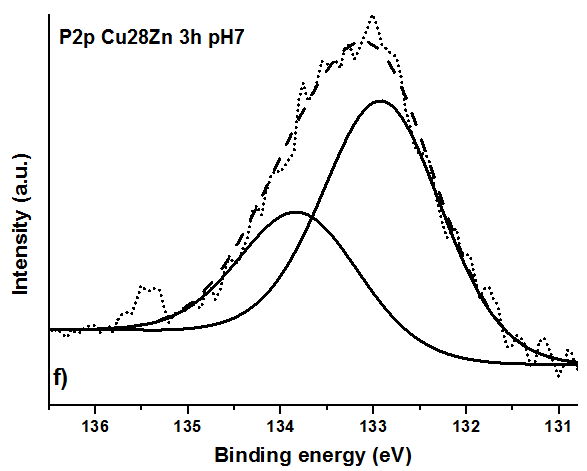


**Figure S.9**: Cu 2p_3/2_ (a), Cu L_3_M_45_M_45_ (b), Zn 2p_3/2_ (c), Zn L_3_M_45_M_45_ (d), O 1s (e), P 2p (f) signals for Cu28Zn after 3 hours of contact with the phosphate buffer solution.


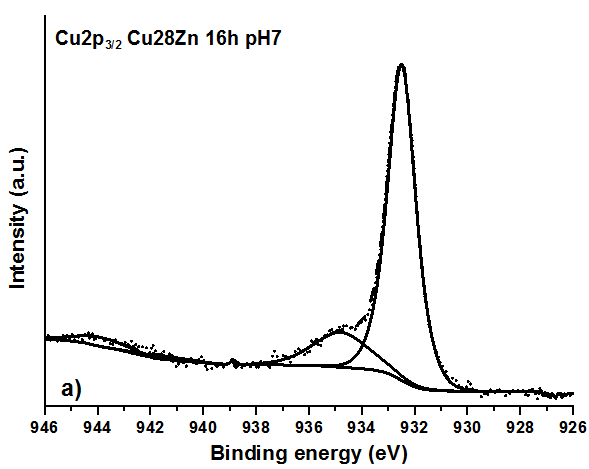

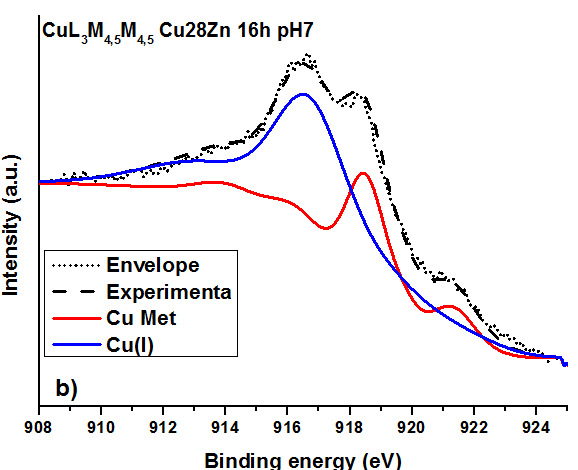

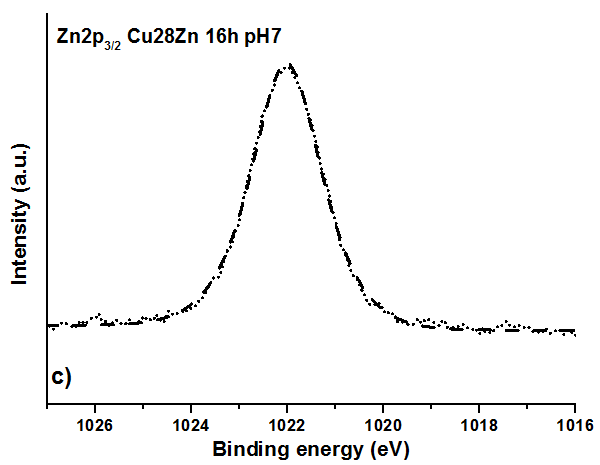

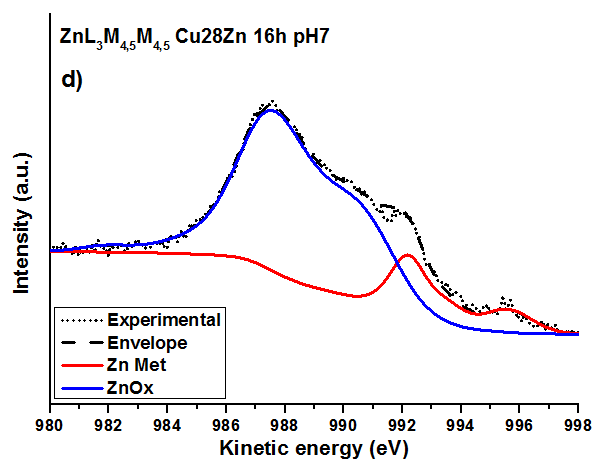

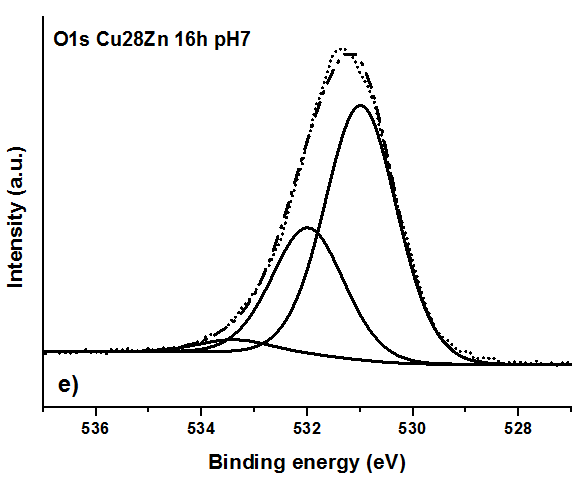

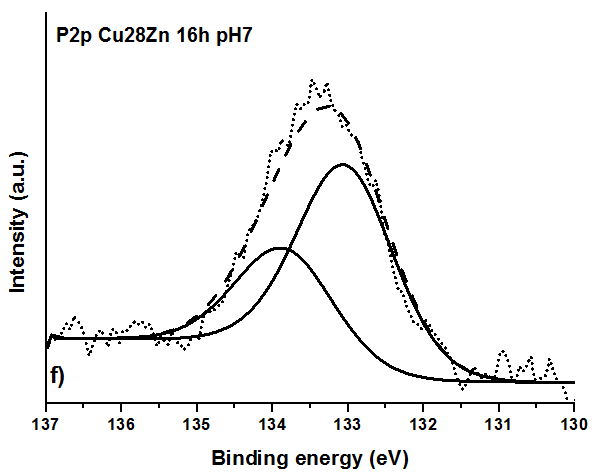


**Figure S.10:** Cu 2p_3/2_ (a), Cu L_3_M_45_M_45_ (b), Zn 2p_3/2_ (c), Zn L_3_M_45_M_45_ (d), O 1s (e), P 2p (f) signals for Cu28Zn after 16 hours of contact with the phosphate buffer solution.


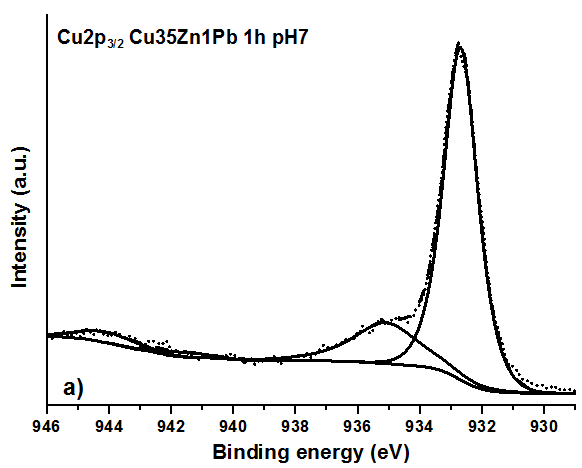

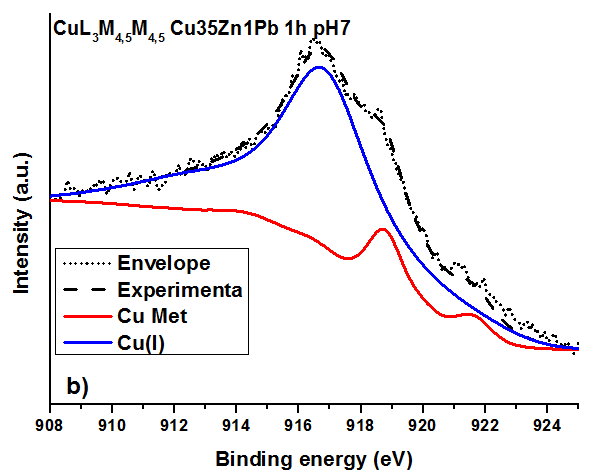

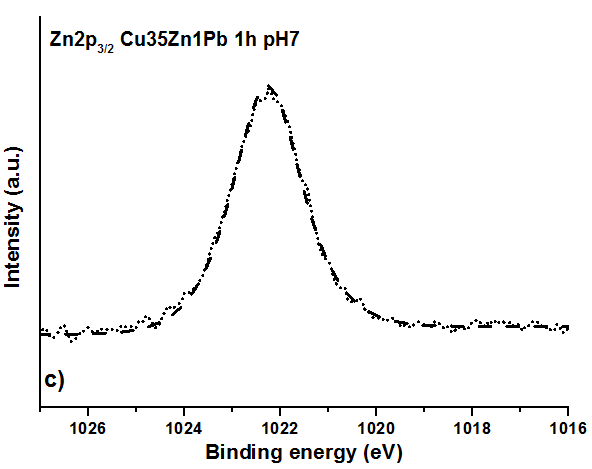

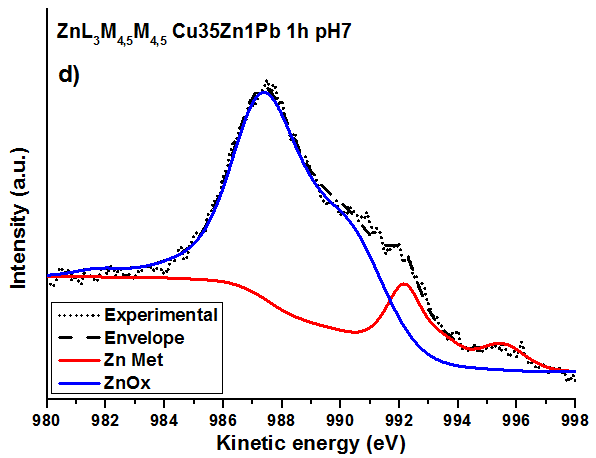

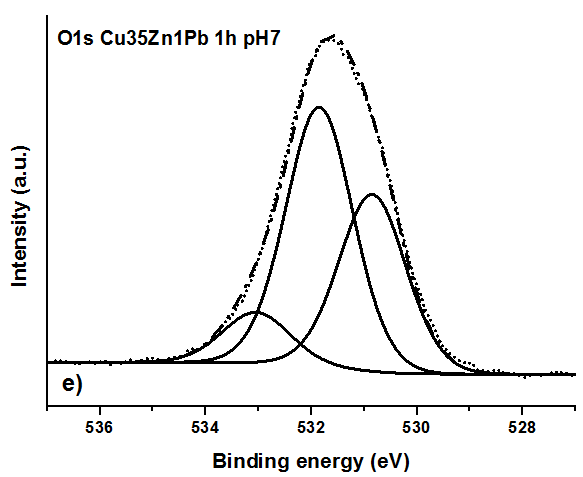

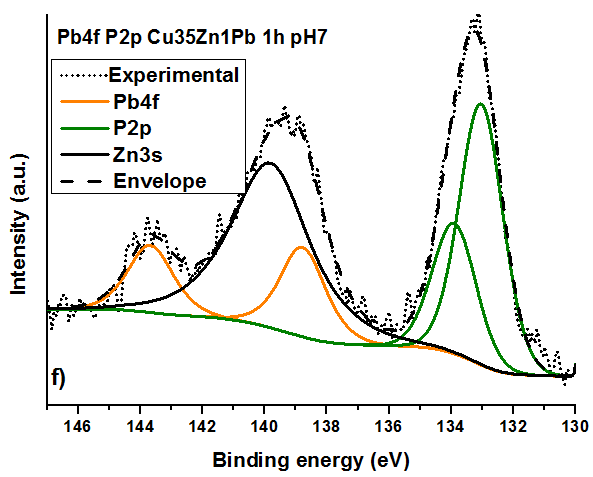


**Figure S.11:** Cu 2p_3/2_ (a), Cu L_3_M_45_M_45_ (b), Zn 2p_3/2_ (c), Zn L_3_M_45_M_45_ (d), O 1s (e), P 2p (f) signals for the Cu35Zn1Pb after 1 hour of contact with the phosphate buffer solution.


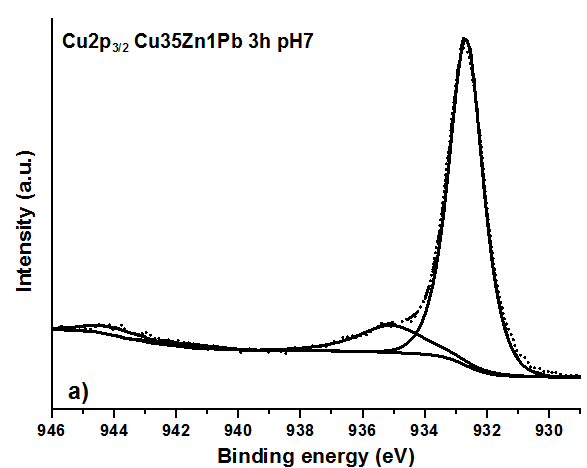

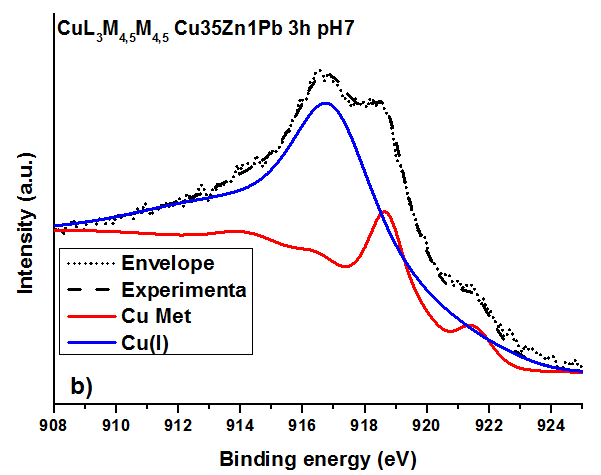

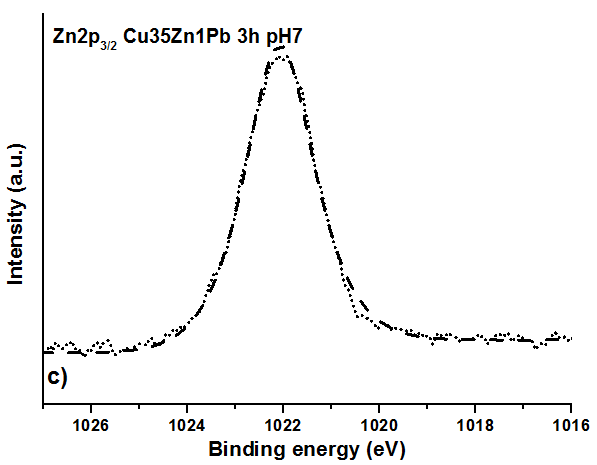

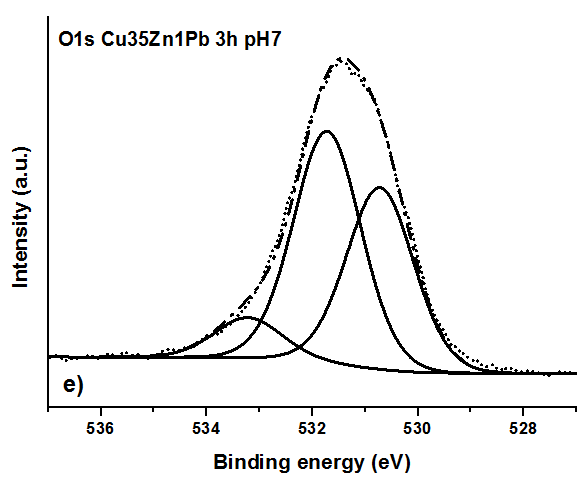

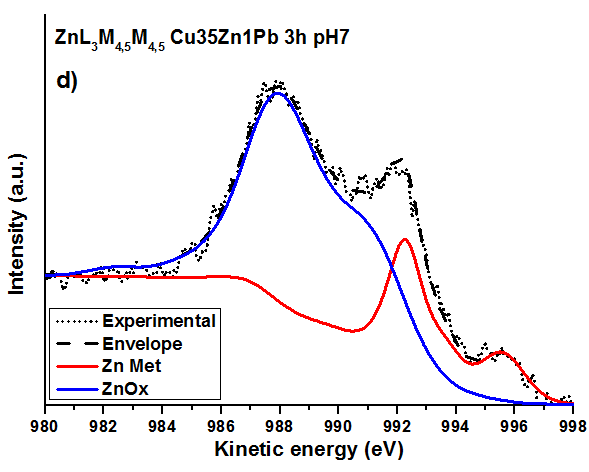

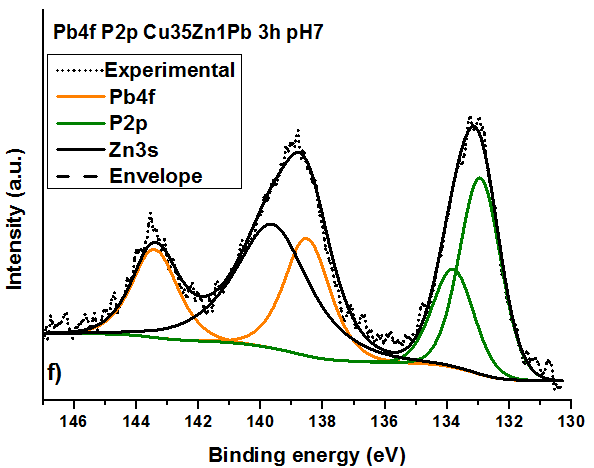


**Figure S.12:** Cu 2p_3/2_ (a), Cu L_3_M_45_M_45_ (b), Zn 2p_3/2_ (c), Zn L_3_M_45_M_45_ (d), O 1s (e), P 2p (f) signals for the Cu35Zn1Pb after 3 hours of contact with the phosphate buffer solution.


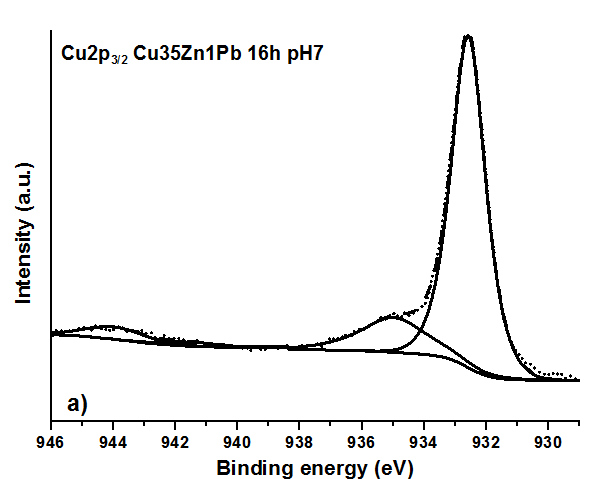

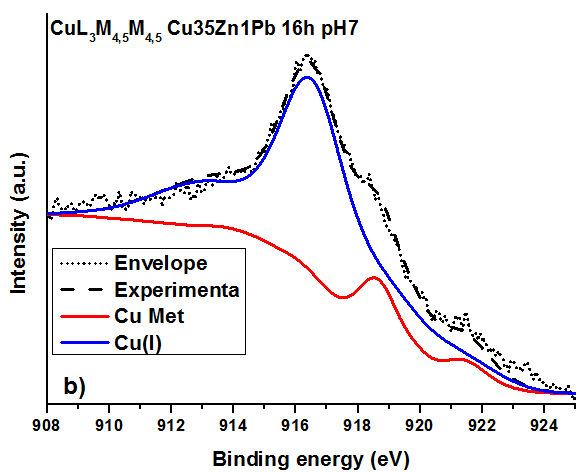

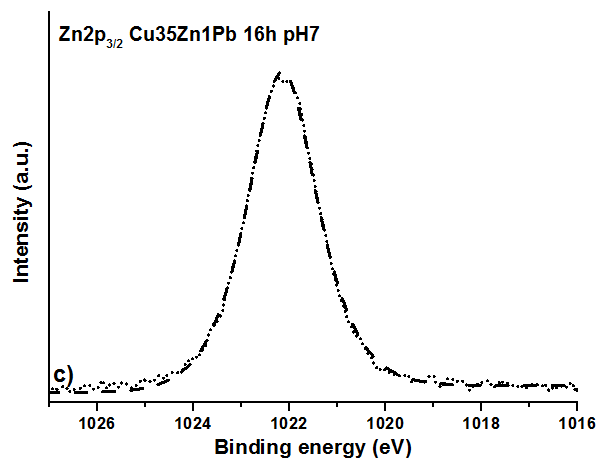

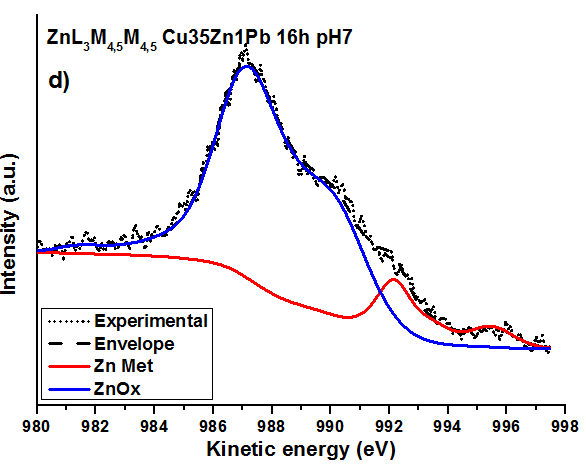

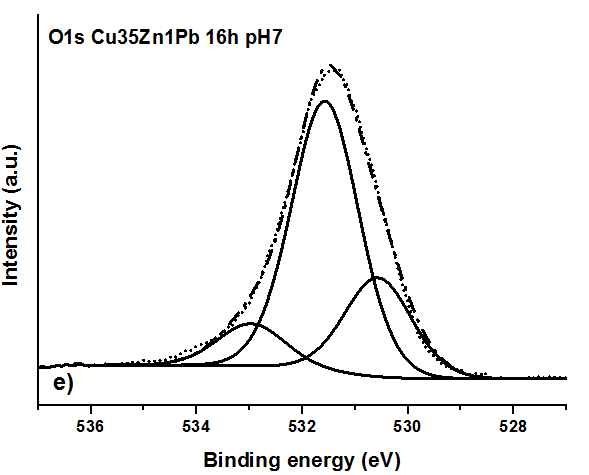

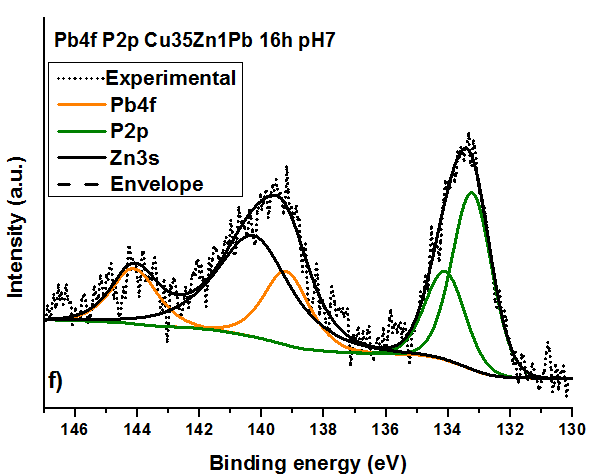


**Figure S.13:** Cu 2p_3/2_ (a), Cu L_3_M_45_M_45_ (b), Zn 2p_3/2_ (c), Zn L_3_M_45_M_45_ (d), O 1s (e), P 2p (f) signals for the Cu35Zn1Pb after 16 hours of contact with the phosphate buffer solution.


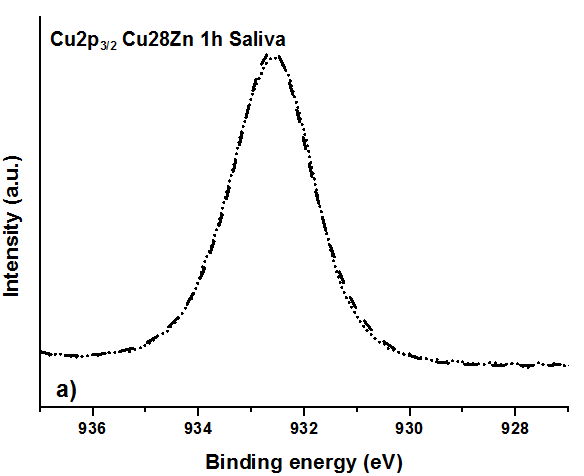

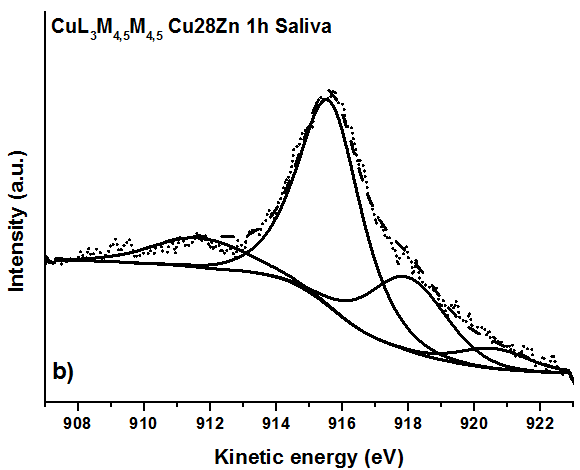

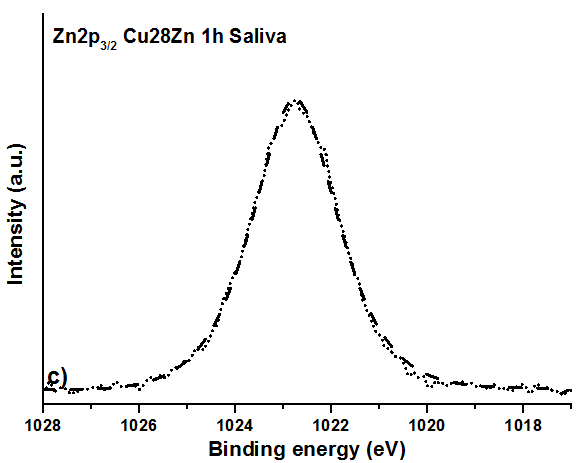

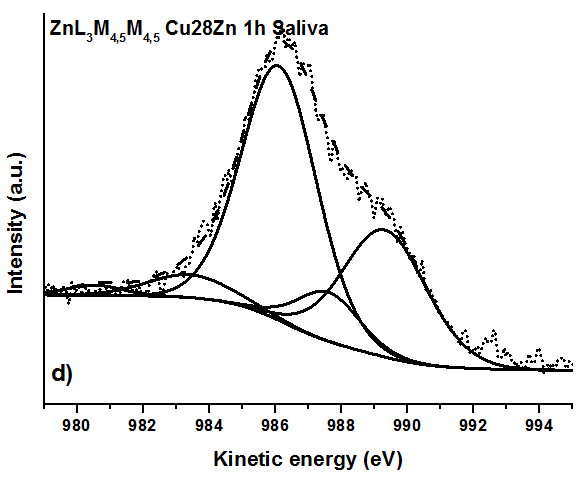

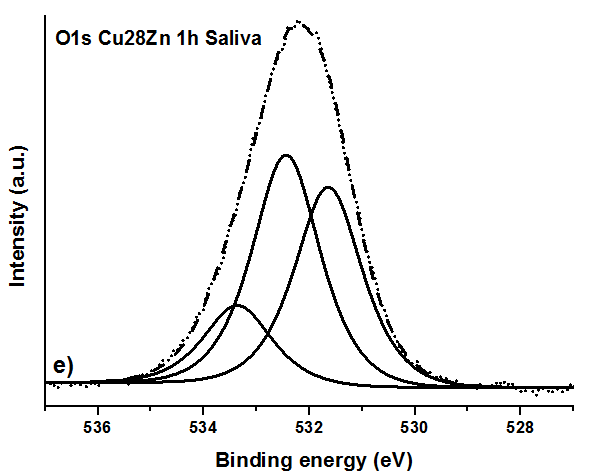

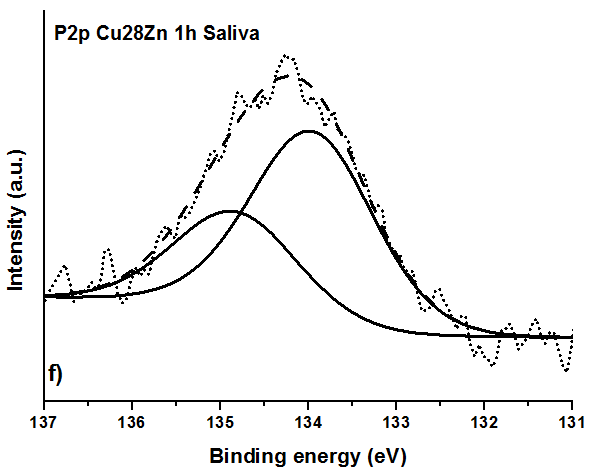

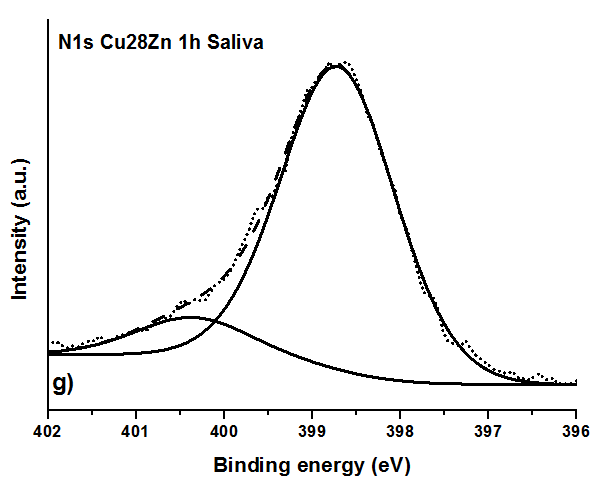

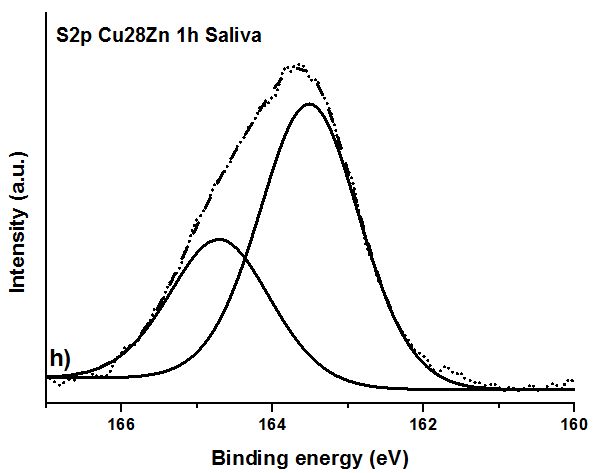


**Figure S.14:** High resolution spectra of Cu 2p_3/2_ (a), Zn 2p_3/2_ (b), O 1s (c), Cu L_3_M_45_M_45_ (d), Zn L_3_M_45_M_45_ (e), P 2p (f), N 1s (g), and S 2p (h) for the Cu28Zn after 1 hour of contact with the saliva solution.


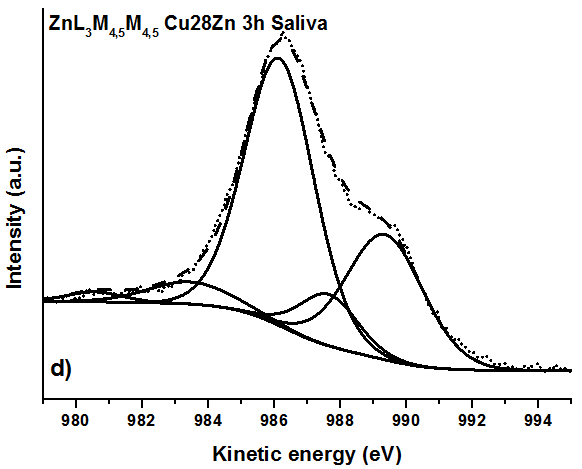

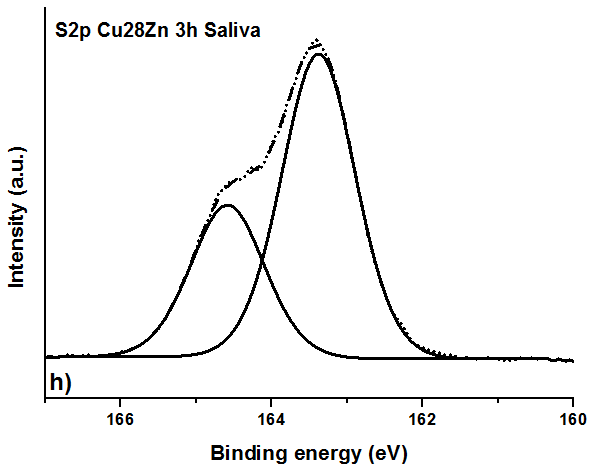

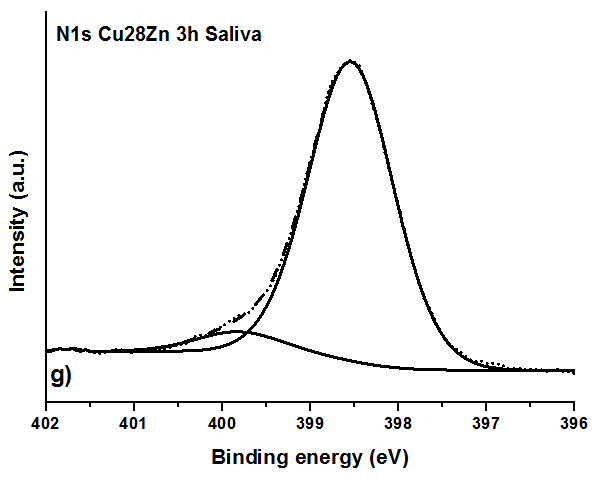

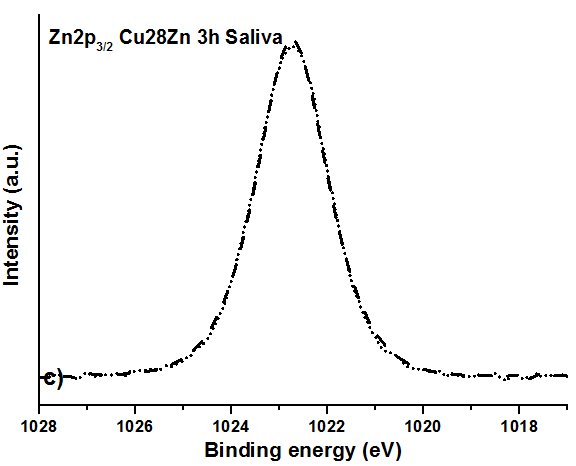

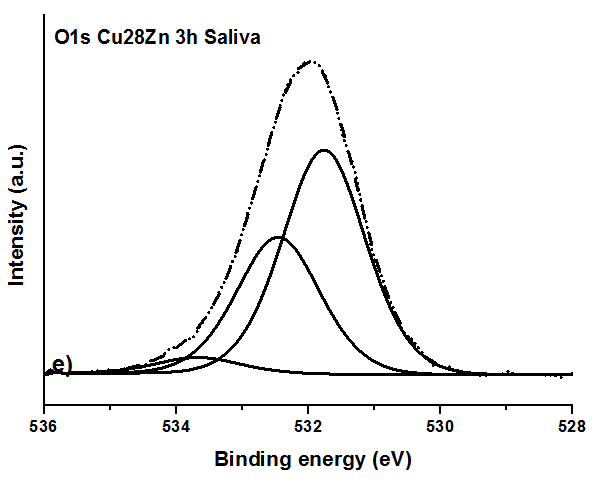

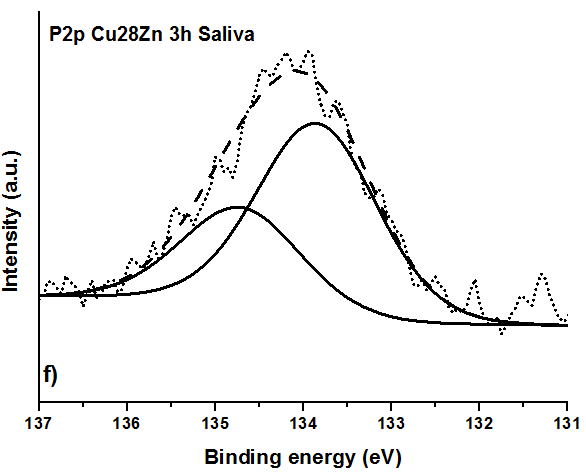


**Figure S.15:** High resolution spectra of Cu 2p_3/2_ (a), Zn 2p_3/2_ (b), O 1s (c), Cu L_3_M_45_M_45_ (d), Zn L_3_M_45_M_45_ (e), P 2p (f), N 1s (g), and S 2p (h) for the Cu28Zn after 3 hours of contact with the saliva solution.


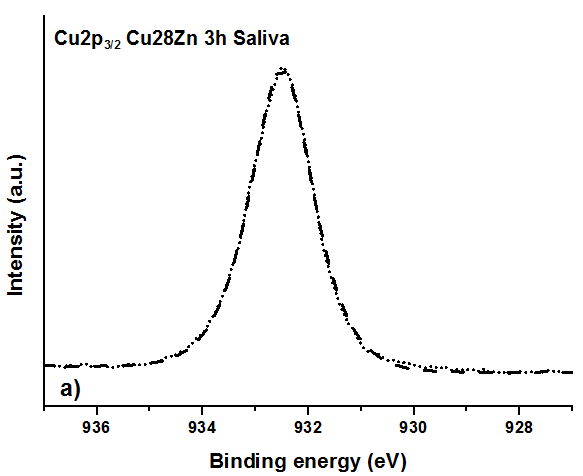

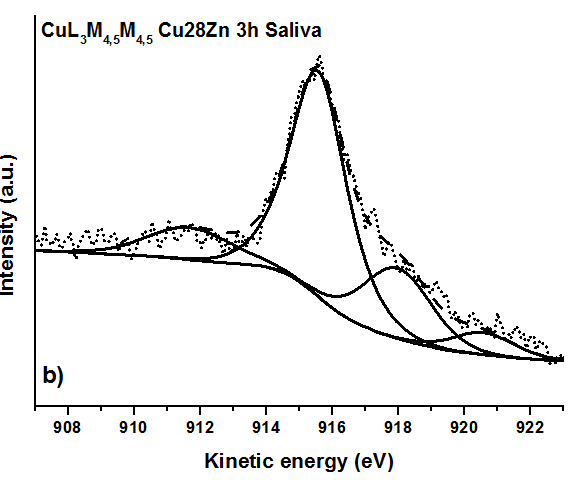

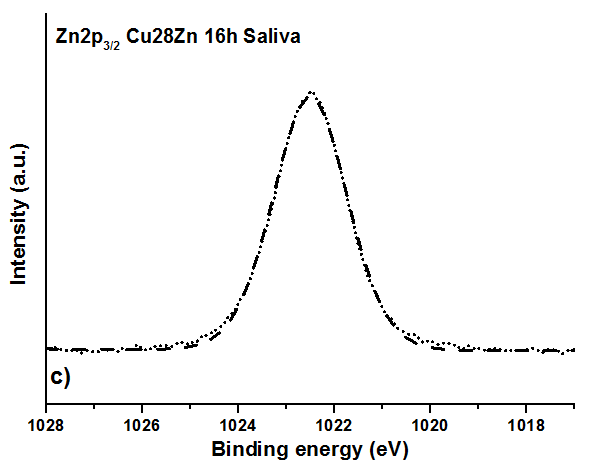

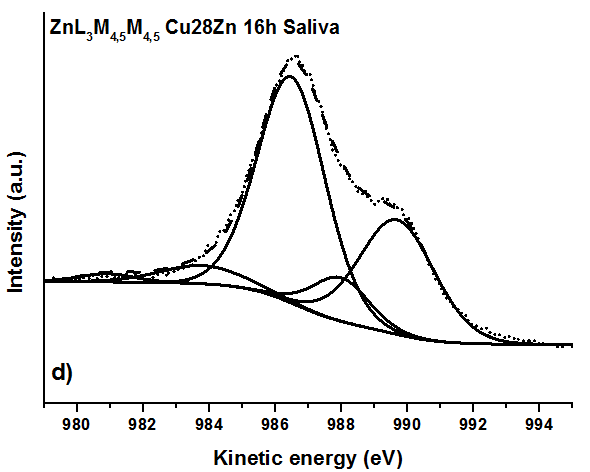

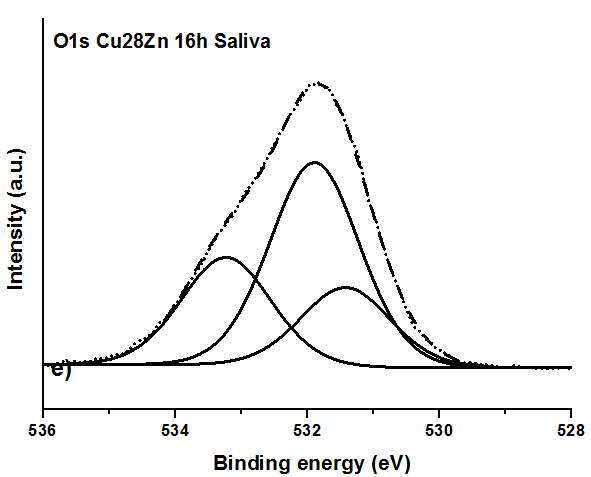

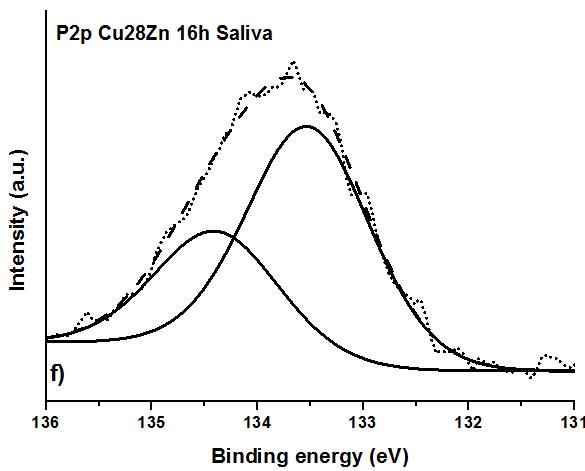

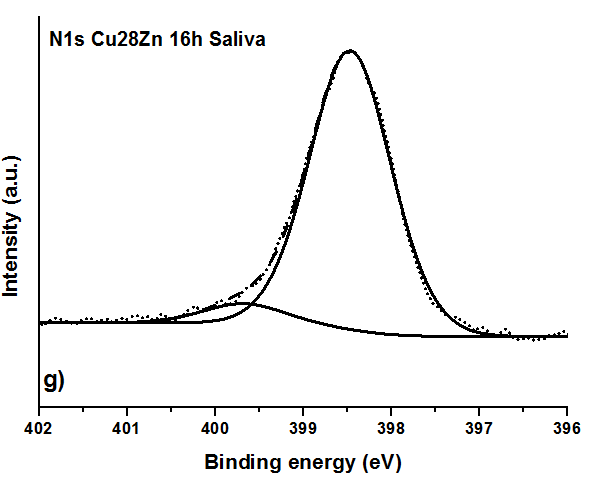

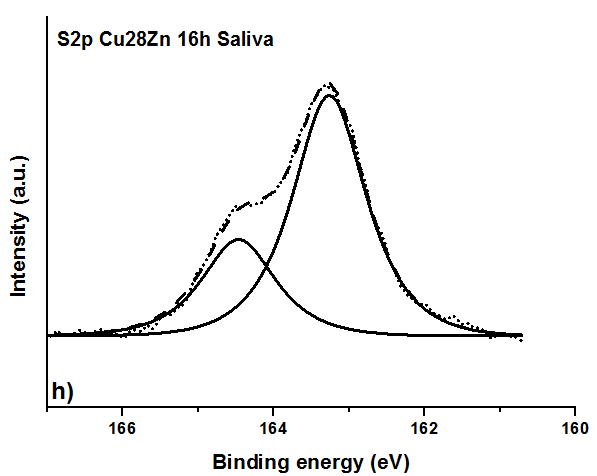


**Figure S.16**: High resolution spectra of Cu 2p_3/2_ (a), Zn 2p_3/2_ (b), O 1s (c), Cu L_3_M_45_M_45_ (d), Zn L_3_M_45_M_45_ (e), P 2p (f), N 1s (g), and S 2p (h) for the Cu28Zn after 16 hours of contact with the saliva solution.

**2.2 Tables**

**Table S.1:** Average binding energy (BE) of the most intense photoelectron peaks, quantitative composition (at %) of the main elements detected on Cu28Zn after exposure to the phosphate buffer solution, and kinetic energy of Auger peaks. The thickness of the contamination layer (l_c_) and of the surface layer (t), together with the composition of the surface layer and of the bulk/surface layer interface, estimated by three layer model [1, 2] are provided. Standard deviations are given in parentheses.

| **Cu28Zn** | **1h pH7** | | | **3h pH7** | | | **16h pH7** | | |
| --- | --- | --- | --- | --- | --- | --- | --- | --- | --- |
|  | **BE (eV)** | | **at%** | **BE (eV)** | | **at%** | **BE (eV)** | | **at%** |
| **Cu 2p_3/2_ Cu(0)** | 932.6 (0.1) | | 5 (2) | 932.5 (0.1) | | 1.4 (0.5) | 932.5 (0.1) | | 6 (3) |
| **Cu 2p_3/2_ Cu(I)** | 932.6 (0.1) | | 9 (3) | 932.5 (0.1) | | 10 (3) | 932.5 (0.1) | | 9 (3) |
| **Cu 2p_3/2_ Cu(II)** | 934.9 (0.1) | | 7 (2) | 934.9 (0.1) | | 6 (2) | 934.8 (0.1) | | 3.0 (0.4) |
| **Sat 1** | 944.0 (0.1) | |  | 943.9 (0.1) | |  | 943.9 (0.1) | |  |
| **Sat 2** | 941.5 (0.1) | |  | 941.4 (0.1) | |  | 941.4 (0.1) | |  |
| **O 1s** | 530.8 (0.1) | | 62 (6) | 530.7 (0.1) | | 61 (6) | 530.8 (0.1) | | 62 (9) |
|  | 532.0 (0.2) | |  | 531.7 (0.1) | |  | 531.8 (0.1) | |  |
|  | 533.5 (0.2) | |  | 533.1 (0.1) | |  | 533.2 (0.1) | |  |
| **P 2p** | 133.2 (0.1) | | 7 (3) | 133.2 (0.2) | | 11 (1) | 133.0 (0.2) | | 12 (1) |
|  | 134.1 (0.1) | |  | 134.0 (0.2) | |  | 133.8 (0.2) | |  |
| **Zn 2p_3/2_ ~~Cu~~ Zn(0)** | 1022.1 (0.1) | | 3.0 (0.4) | 1022.2 (0.1) | | 2.0 (0.2) | 1022.0 (0.1) | | 4.0 (0.6) |
| **Zn 2p_3/2_ Zn(II)** | 1022.1 (0.1) | | 7 (3) | 1022.2 (0.1) | | 8 (2) | 1022.0 (0.1) | | 5 (1) |
|  |  | |  |  | |  |  | |  |
|  | **KE (eV)** | | | **KE (eV)** | | | **KE (eV)** | | |
| **Cu L_3_M_4,5_M_4,5_ met** | 918.5 (0.1) | | | 918.6 (0.1) | | | 918.5 (0.1) | | |
| **Cu L_3_M_4,5_M_4,5_Ox** | 916.5 (0.2) | | | 916.9 (0.2) | | | 916.5 (0.2) | | |
|  | **KE (eV)** | | | **KE (eV)** | | | **KE (eV)** | | |
| **Zn L_3_M_4,5_M_4,5_met** | 992.1 (0.1) | | | 992.3 (0.1) | | | 992.2 (0.1) | | |
| **Zn L_3_M_4,5_M_4,5_Ox** | 987.3 (0.3) | | | 987.2 (0.1) | | | 987.5 (0.1) | | |
| **Thickness of the layers and composition of oxide layer and of the interface bulk/oxide layer** | | | | | | | | | |
| **l_c_** | | 1.3 (0.2) nm | | | 1.9 (0.2) nm | | | 1.11 (0.04) nm | |
| **t** | | 1.1 (0.1) nm | | | 0.8 (0.1) nm | | | 1.60 (0.04) nm | |
| **Oxide layer** | | Cu-ox = 63 (12) %  Zn-ox = 37 (12) % | | | Cu-ox = 63 (6) %  Zn-ox = 37 (6) % | | | Cu – ox = 65 (5) %  Zn-ox 35 (5) % | |
| **Oxide/bulk interface** | | Cu met = 58 (4) %  Zn met = 42 (4) % | | | Cu met = 32 (10) %  Zn met = 68 (10) % | | | Cu met = 55 (75 %  Zn met = 45 (5) % | |

**Table S.2:** Average binding energy (BE) of the most intense photoelectron peaks, quantitative composition (at %) of the main elements detected on Cu35Zn1Pb after exposure to the phosphate buffer solution, and kinetic energy of Auger peaks. The thicknesses of the contamination layer (lc) and of the surface layer (t), together with the composition of the surface layer and of the bulk/surface layer interface, estimated by three-layer model [1, 2] are provided. Standard deviations are given in parentheses.

| **Cu35Zn1Pb** | **1h pH7** | | | **3h pH7** | | | **16h pH7** | | |
| --- | --- | --- | --- | --- | --- | --- | --- | --- | --- |
|  | **BE (eV)** | | **at %** | **BE (eV)** | | **at %** | **BE (eV)** | | **at %** |
| **Cu 2p_3/2_ Cu(0)** | 932.6 (0.1) | | 3.2 (0.5) | 932.7 (0.1) | | 6 (2) | 932.6 (0.1) | | 0.7 (0.3) |
| **Cu 2p_3/2_ Cu(I)** | 932.6 (0.1) | | 12 (2) | 932.7 (0.1) | | 16 (3) | 932.6 (0.1) | | 5 (1) |
| **Cu 2p_3/2_ Cu(II)** | 935.0 (0.1) | | 4.4 (0.3) | 935.1 (0.1) | | 4 (1) | 935.0 (0.1) | | 5 (1) |
| **Sat 1** | 944.0 (0.1) | |  | 944.1 (0.1) | |  | 944.0 (0.1) | |  |
| **Sat 2** | 941.5 (0.1) | |  | 941.6 (0.1) | |  | 941.5 (0.1) | |  |
| **O 1s** | 530.8 (0.1) | | 42 (4) | 530.7 (0.1) | | 57 (5) | 530.7 (0.1) | | 59 (4) |
|  | 531.8 (0.1) | |  | 531.7 (0.1) | |  | 531.7 (0.1) | |  |
|  | 533.0 (0.1) | |  | 533.2 (0.1) | |  | 533.1 (0.1) | |  |
| **Pb 4f Pb-Ox** | 138.8 (0.1) | | 0.3 (0.1) | 138.7 (0.1) | | 0.3 (0.1) | 138.9 (0.1) | | 0.10 (0.01) |
|  | 143.7 (0.1) | |  | 143.6 (0.1) | |  | 144.1 (0.1) | |  |
| **P 2p** | 133.0 (0.1) | | 12 (2) | 133.0 (0.2) | | 10 (2) | 133.2 (0.1) | | 16 (2) |
|  | 133.8 (0.1) | |  | 133.8 (0.2) | |  | 134.1 (0.1) | |  |
| **Zn 2p_3/2_ ~~Cu~~ Zn(0)** | 1022.2 (0.1) | | 2.2 (0.1) | 1022.1 (0.1) | | 3 (1) | 1022.2 (0.1) | | 1.3 (0.3) |
| **Zn 2p_3/2_ Zn(II)** | 1022.2 (0.1) | | 4.2 (0.2) | 1022.1 (0.1) | | 4.0 (0.5) | 1022.2 (0.1) | | 13 (1) |
|  | | | | | | | | | |
|  | **KE (eV)** | | | **KE (eV)** | | | **KE (eV)** | | |
| **Cu LMM met** | 918.8 (0.1) | | | 918.7 (0.1) | | | 918.7 (0.1) | | |
| **Cu LMM Ox** | 916.9 (0.1) | | | 916.9 (0.1) | | | 916.6 (0.1) | | |
|  | | | | | | | | | |
|  | **KE (eV)** | | | **KE (eV)** | | | **KE (eV)** | | |
| **Zn LMM met** | 992.3 (0.1) | | | 992.3 (0.1) | | | 992.2 (0.1) | | |
| **Zn LMM Ox** | 987.4 (0.1) | | | 987.8 (0.1) | | | 987.2 (0.1) | | |
| **Thickness of the layers and composition of oxide layer and of the interface bulk/oxide layer** | | | | | | | | | |
| **l_c_** | | 1.58 (0.03) nm | | | 1.4 (0.1) nm | | |  | |
| **t** | | 1.00 (0.03) nm | | | 0.72 (0.07) nm | | |  | |
| **Oxide layer** | | Cu-ox = 78 (1) %  Zn-ox = 22 (1) % | | | Cu-ox = 82 (2) %  Zn-ox = 18 (2) % | | |  | |
| **Oxide/bulk interface** | | Cu met = 48 (7) %  Zn met = 52 (7) % | | | Cu met = 52 (8) %  Zn met = 48 (8) % | | |  | |

**Table S.3:** Average binding energy (BE) of the most intense photoelectron peaks, quantitative composition (at %) of the main elements detected on Cu28Zn after exposure to the saliva solution. Standard deviations are given in parentheses.

| **Cu28Zn** | **1h** | | **3h** | | **16h** | |
| --- | --- | --- | --- | --- | --- | --- |
|  | **BE (eV)** | **at %** | **BE (eV)** | **at %** | **BE (eV)** | **at %** |
| **Cu** | 932.6 (0.1) | 23 (1) | 932.6 (0.1) | 13 (3) | 932.6 (0.1) | 12 (2) |
| **N 1s** | 398.7 (0.1) | 16 (1) | 398.5 (0.1) | 9 (2) | 398.4 (0.1) | 8 (1) |
| **N NCS** | 400.1 (0.1) |  | 399.9 (0.1) |  | 399.7 (0.1) |  |
| **O 1** | 531.6 (0.1) | 27 (1) | 531.8 (0.1) | 36 (7) | 531.4 (0.1) | 45 (2) |
| **O 1s** | 532.5 (0.1) |  | 532.5 (0.1) |  | 533.2 (0.1) |  |
| **O 1s** | 533.5 (0.2) |  | 533.7 (0.1) |  | 531.9 (0.1) |  |
| **P 2p** | 134.0 (0.1) | 5 (1) | 133.8 (0.1) | 9 (2) | 133.8 (0.1) | 9 (1) |
| **P 2p** | 134.8 (0.1) |  | 134.7 (0.1) |  | 134.3 (0.1) |  |
| **S 2p_3/2_ SCN** | 163.5 (0.1) | 19 (1) | 163.3 (0.1) | 16 (2) | 163.2 (0.1) | 12 (2) |
| **S 2p_1/2_** | 164.7 (0.1) |  | 164.6 (0.1) |  | 164.4 (0.1) |  |
| **Zn 2p** | 1022.8 (0.1) | 10 (1) | 1022.8 (0.1) | 16 (2) | 1022.7 (0.1) | 14 (2) |
|  | **KE (eV)** | | **KE (eV)** | | **KE (eV)** | |
| **Cu LMM** | 915.7 (0.1) | | 915.7 (0.1) | | 915.8 (0.1) | |
| **Zn LMM** | 986.5 (0.1) | | 986.4 (0.1) | | 986.6 (0.1) | |

**Reference**

# Cocco. F., Fantauzzi, M., Elsener, B., Atzei, D., Rossi, A. (2016). [Nanosized surface films on brass alloys by XPS and XAES](https://www.scopus.com/record/display.uri?eid=2-s2.0-84962016049&origin=resultslist&sort=plf-f&src=s&sid=6bf38415d333a1a1efac446bb1c821d2&sot=autdocs&sdt=autdocs&sl=18&s=AU-ID%2856954794100%29&relpos=2&citeCnt=8&searchTerm=); *RSC Advances* 6, 31277-31289. DOI: 10.1039/C5RA23135C

# Rossi and Elsener XPS analysis of passive films on the amorphous alloy Fe70Cr10P13C7: Effect of the applied potential; *Surf. Interface Anal.*, 1992, 18 , 499-504 <https://doi.org/10.1002/sia.740180708>
